# Nitric Oxide Tunes Secreted Metabolite Bioactivity

**DOI:** 10.1111/mmi.70083

**Published:** 2026-06-08

**Authors:** Zachery R. Lonergan, Sarah L. Weisflog, Matthew Scurria, Jinyang Li, Korbinian Thalhammer, Osvaldo Gutierrez, Stuart J. Conway, Dianne K. Newman

**Affiliations:** ^1^ Division of Biology and Biological Engineering California Institute of Technology Pasadena California USA; ^2^ Department of Biochemistry and Microbiology Rutgers University New Brunswick New Jersey USA; ^3^ Department of Chemistry and Biochemistry University of California Los Angeles California USA; ^4^ Division of Geological and Planetary Sciences California Institute of Technology Pasadena California USA

**Keywords:** nitric oxide, phenazine, *pseudomonas*, pyocyanin

## Abstract

The radical nitric oxide (·NO) is short‐lived but has imprinted itself on many aspects of physiology and disease. ·NO's rapid production and consumption, coupled with its intrinsic reactivity, drive its biological importance; thus, defining mechanisms and targets of ·NO reactivity is necessary to assess its fate and impact. Cellular small molecules are a major class of ·NO‐reactive targets, possessing a variety of molecular functionalities that can react with ·NO. Yet the capacity for secreted small molecules to react with ·NO, as well as the biological consequences of such reactivity, have received little attention. Here, we explore the reactivity of ·NO with phenazine metabolites, microbially‐derived secreted small molecules that possess antibiotic properties and can modulate their microenvironment. Using *Pseudomonas aeruginosa* as a model phenazine producer, we find that ·NO reacts with specific phenazines to yield stable, chemically distinct products. These chemical transformations significantly attenuate phenazine antibiotic properties, including against the phenazine nonproducer *Staphylococcus aureus* , a competitor with *P. aeruginosa* for niches in the context of infection. By contrast, *P. aeruginosa* experiences rapid loss in viability when phenazines and ·NO react. This toxicity occurs even in the presence of *S. aureus* , which displays resistance to nitrosylated phenazines, implicating a specific toxicity dependent on the formation of the phenazine‐NO adduct. These findings highlight the capacity of ·NO to transform metabolite activity and suggest that ·NO can tune microbial interactions in complex environments by a mechanism of action hitherto unappreciated.

## Graphical abstract

*Pseudomonas aeruginosa* produces phenazine metabolites that can react with nitric oxide (·NO) to form nitrosylated compounds. The formation of these metabolites results in rapid loss in *P. aeruginosa* viability and diminishes phenazine antimicrobial activity against other organisms, including *Staphylococcus aureus* .

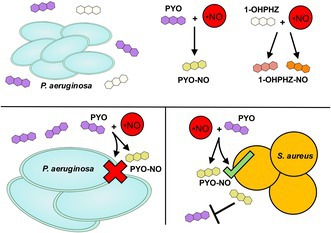

## 1 Introduction

Reactive oxygen and nitrogen species are produced continuously in many biological systems. Among these is the radical nitric oxide (·NO), which is widely recognized as a key component of human physiology as a regulator of neurotransmission, vascular dilation, and infection control (Lundberg and Weitzberg 2022). NO also plays crucial functions for microbial physiology, including as an intermediate during anaerobic respiration and as an inter‐ and intracellular signaling molecule (Chen et al. 2022). The widespread importance of ·NO stems from its intrinsic reactivity coupled with its rapid diffusion, which allows broad‐acting effects from a focal point of production over short timescales (Lundberg and Weitzberg 2022). ·NO can also react with oxygen and superoxide to yield other reactive nitrogen species, including nitroxyl radicals, nitrogen dioxide, and peroxynitrite, which expand its impact indirectly (Squadrito and Pryor 1998; Szabo et al. 2007).

While ·NO is commonly described as highly reactive, there is specificity to its chemistry that is intimately linked to its existence as a radical (Wink and Mitchell 1998). ·NO preferentially reacts with specific types of molecules and motifs, including phenolic compounds, thiols, amines, and transition metal centers (Crow and Beckman 1995; Gaston 1999; Ford and Lorkovic 2002). These molecular features are common components of proteins, and significant progress has been made in discerning how ·NO‐protein reactivity can alter structure–function relationships (Gow et al. 2004; Hess et al. 2005). However, these ·NO‐reactive motifs are also ubiquitous in the small molecule landscape (Baran et al. 2007). Well‐described examples demonstrating ·NO‐small molecule reactivity include ·NO scavenging via the antioxidant glutathione and ·NO reactivity with the amino acids tyrosine and cysteine (Hogg et al. 1996; Ischiropoulos 1998; Wink et al. 1994), sugars such as glucose (Brodsky et al. 2001), and fatty acids (O'Donnell et al. 1999). However, the small molecule landscape is both vast and chemically complex, with ~10^60^ small molecules theoretically possible (Ohlmeyer and Zhou 2010). Given their structural diversity and environmental ubiquity, the capacity for ·NO to react with small molecules merits more attention.

Metabolites that are secreted into the environment are common yet often overlooked with respect to their potential to react with ·NO. Like ·NO, secreted small molecules can modify the local chemical environment via their diffusive capacity (Camilli and Bassler 2006; Dobson 2004). An important class of secreted small molecules are phenazine metabolites, whose production has been documented in environments ranging from soils to human chronic infections (Mavrodi et al. 2010; Price‐Whelan et al. 2006; Muller et al. 2009). One prolific phenazine producer is the opportunistic bacterial pathogen *Pseudmonas aeruginosa* that produces the well‐studied molecule pyocyanin (PYO), as well as three additional phenazines including 1‐hydroxyphenazine (1‐OHPHZ), phenazine‐1‐carboxylic acid (PCA), and phenazine‐1‐carboxyamide (PCN) (Mavrodi et al. 2001). Phenazine production plays important roles in *P. aeruginosa* virulence by supporting bacterial anaerobic survival and intoxicating immune cells (Wang et al. 2010; Glasser et al. 2014; Bianchi et al. 2008), but phenazines also possess antimicrobial activity, thus serving as natural antibiotics (Price‐Whelan et al. 2006; Lau et al. 2004; Thomashow and Weller 1988). PYO and other phenazines possess structural features consistent with known ·NO‐reactive molecules and are produced in infection environments where ·NO concentrations can be high due to release from innate immune cells (Caldwell et al. 2009; Allen et al. 2005), but the ability of phenazines to react with ·NO is unclear and disputed (Warren et al. 1990; Vukomanovic et al. 1997; Gusarov et al. 2009). We therefore sought to investigate ·NO‐phenazine reactivity to clarify the chemistry governing these interactions as an example of how secreted metabolites might impact ·NO fate and biological consequences in disease contexts.

## 2 Results

### 2.1 Pyocyanin and Nitric Oxide Alter *P. aeruginosa* Growth and Metabolite Detection

**FIGURE 1:**
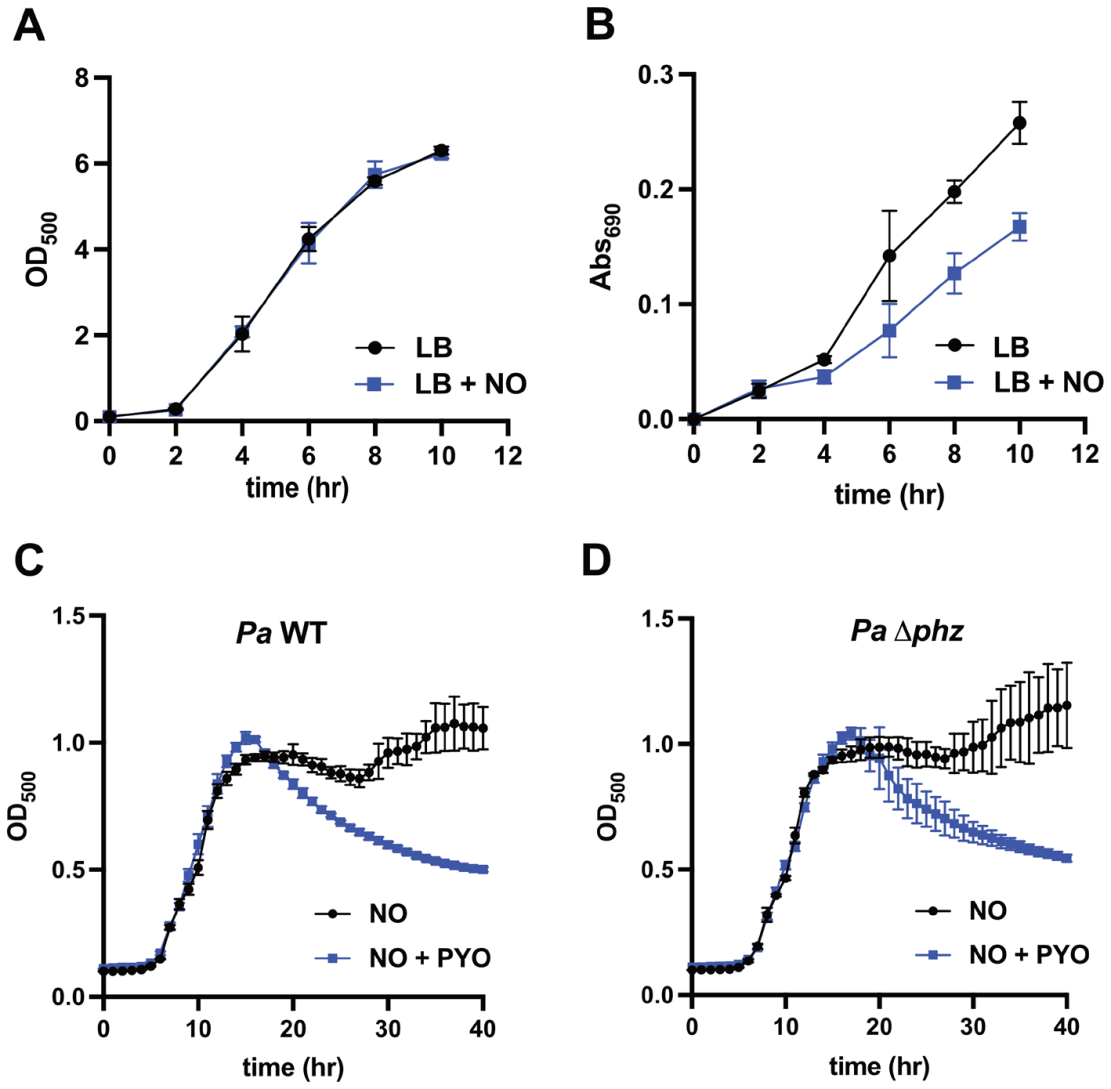
Nitric oxide exposure reduces pyocyanin detection in *P. aeruginosa* . (A) Growth monitored by absorbance at 500 nm of *P. aeruginosa* (*Pa*) in lysogeny broth (LB) supplemented with 1 mM DETA‐NONOate ·NO donor; *n* = 3 biological replicates, mean ± standard deviation (SD). (B) Relative abundance of pyocyanin (PYO) in supernatant measured via absorbance at 690 nm at times matching (A); *n* = 3 biological replicates, mean ± SD. (C) Representative growth monitored by absorbance at 500 nm of wildtype (WT) *Pa* with 1 mM DETA‐NONOate and 100 μM PYO supplemented at the start of growth; *n* = 3 technical replicates from 1 of 3 biological replicates with mean ± SD. (D) Representative growth monitored by absorbance at 500 nm of Δ*phz Pa* with 1 mM DETA‐NONOate and 100 μM PYO supplemented at the start of growth; *n* = 3 technical replicates from 1 of 3 biological replicates, mean ± SD.

·NO is known to inhibit *P. aeruginosa* growth and cause cell death, but the effect of ·NO on physiology is linked to its concentration. Consistent with previous results (Yoon et al. 2006; Wilbert and Newman 2022), acute exposure to high concentrations of ·NO kills *P. aeruginosa* (Figure S1A), but ·NO also can alter physiology without affecting viability (Barraud et al. 2006). We found that *P. aeruginosa* can grow with low‐level, slow ·NO exposure with no change in the overall cell density (Figure 1A). Growth of *P. aeruginosa* also typically results in a blue‐green pigmentation of the culture, which largely reflects the production of the secreted phenazine metabolite pyocyanin (PYO) (Jayaseelan et al. 2014). Regulation of PYO production is complex, but quorum‐sensing systems play an important role in facilitating increased production of PYO as cell density increases (Dietrich et al. 2006; Haussler and Becker 2008). Consistent with observations from other strains (Gao et al. 2016), PYO detection in the presence of subinhibitory levels of ·NO diminishes over time, without altering growth kinetics or cell density (Figure 1B).

Because PYO detection diminished in the presence of ·NO, we wondered how supplementing growing cultures with excess PYO would further influence *P. aeruginosa* physiology in both a wildtype (WT) strain of *P. aeruginosa* , as well as a phenazine‐biosynthesis mutant strain (Δ*phz*) that cannot produce or modify exogenously‐provided phenazines (Meirelles et al. 2021). Addition of PYO at physiologically‐relevant concentrations does not negatively impact *P. aeruginosa* growth (Figure S1B,C). Supplementation of cultures with ·NO also follows predictable bacterial growth kinetics including a lag phase, exponential growth phase, and stationary phase (Figure 1C,D). The combination of PYO and ·NO permits bacterial growth that follows a similar trajectory in the early phases, but at the transition to stationary phase there is a drop in culture density indicative of cell lysis and loss of viability (Figure 1C,D) (Watt and Clarke 1994). Collectively, these results illustrate that ·NO alters PYO detection and that the co‐occurrence of ·NO and PYO negatively impacts *P. aeruginosa* growth, suggesting that ·NO and PYO might be interacting.

### 2.2 Nitric Oxide Reacts With Phenazines, Yielding Chemically Distinct Products

**FIGURE 2:**
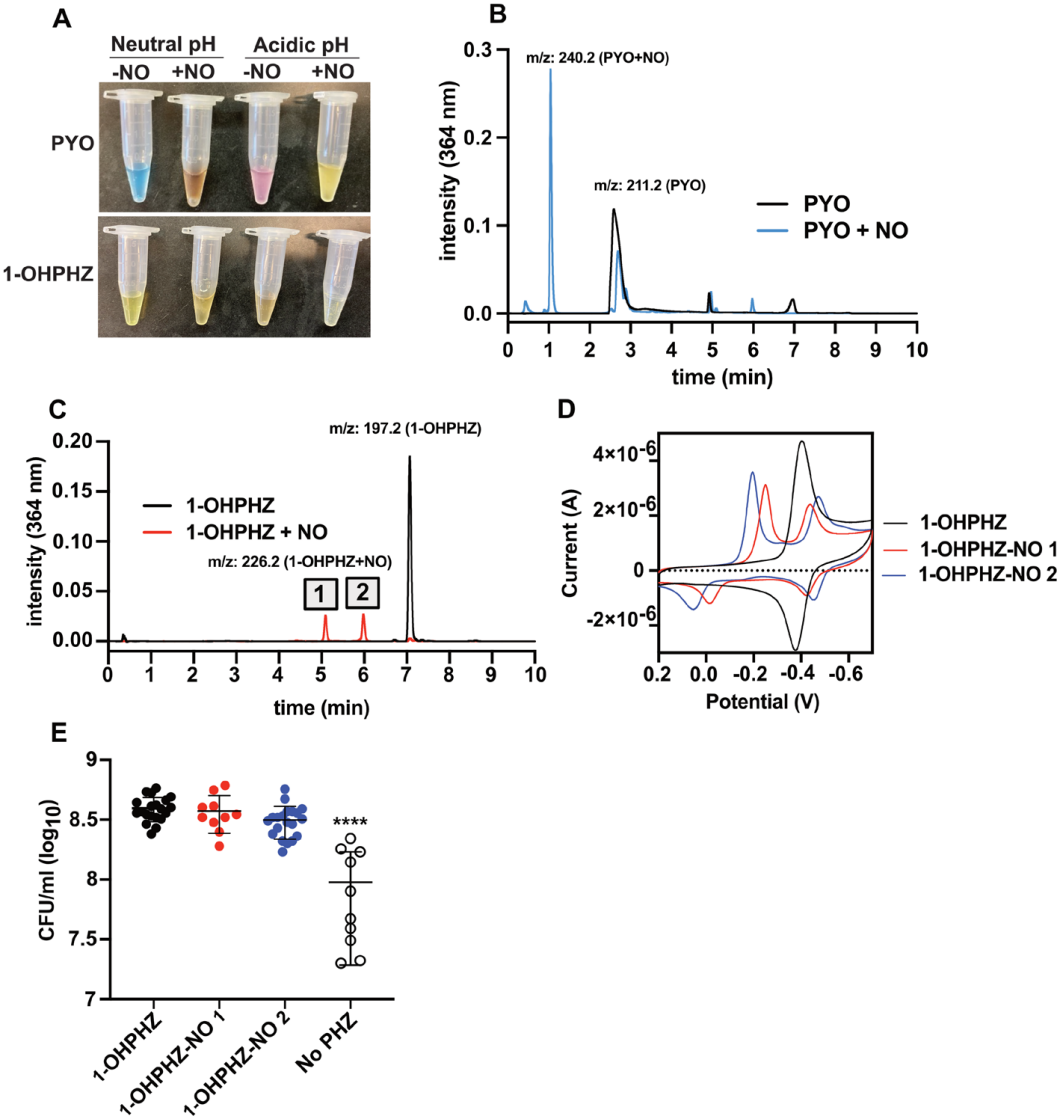
Nitric oxide reacts with phenazines to yield chemically distinct redox‐active metabolites. (A) Image of 500 μM pyocyanin (PYO) or 1‐hydroxyphenazine (1‐OHPHZ) at pH 7 or pH 4 after exposure to ·NO delivered by incubation with 3 mM DEA‐NONOate. (B) Chromatogram for absorbance at 364 nm and MS analyses for phenazines: *m/z* of PYO reacted with ·NO (PYO‐NO) with reported. (C) LC–MS analysis and *m/z* of 1‐OHPHZ reacted with ·NO (1‐OHPHZNO) with absorbance at 364 nm reported and two products labeled #1 and #2. (D) Cyclic voltammetry of 1‐OHPHZ and derivatives. (E) Anaerobic survival after 24 h measured by colony forming units (CFU) per mL of *P. aeruginosa* provided with 100 μM 1‐OHPHZ or its derivatives. ****p < 0.0001, one‐way ANOVA with Tukey multiple comparisons vs. ‘No PHZ;’ *n* = 10–20 from three biological replicates, bars are mean ± SD.

Given the capacity of PYO in the presence of ·NO to alter *P. aeruginosa* growth, we proceeded to investigate the chemical interactions between PYO, ·NO, and phenazines in more detail. *P. aeruginosa* generally produces and secretes four structurally distinct phenazine metabolites that promote its anaerobic survival and are antagonistic against other organisms, including other microbes and humans, and thus can serve as natural antibiotics (Hall et al. 2016; Baron and Rowe 1981). We found that when PYO and the structurally similar 1‐OHPHZ are exposed to ·NO a color change occurs (Figure 2A) and a new product is formed (Table S1). These changes occur in both neutral and acidic conditions (Figure 2A, Figure S2A,B). Spectral analyses after ·NO exposure also suggest absorption properties consistent with phenazine metabolites, most notably an absorbance peak between 300 and 400 nm that is also present in the parent phenazine molecules (Figure S2B,C).

To determine the chemical nature of these ·NO‐exposed phenazines, samples were analyzed using LC–MS, and mass to charge ratios (*m/z*) were compared to the parent molecule. PYO (*m/z* [M + H]^+^ = 211.2) possesses a retention time of ~3 min, while a new product emerges following exposure to ·NO with a retention time of 1 min and mass addition of 29 Da (*m/z* [M + ·NO] = 240.2) (Figure 2B). 1‐OHPHZ (*m/z* 197.2) exposed to ·NO displays two new masses (*m/z* [M + ·NO] = 226.2) with distinct retention times and a mass addition of 29 Da relative to the parent molecule (Figure 2C). These products form over various pH ranges and in both the presence and absence of ambient oxygen (Figure S2C,D; Table S1). The chemical similarities between the PYO and 1‐OHPHZ ·NO‐reacted products suggest a conserved reaction mechanism that might be revealed with additional experimentation with the two 1‐OHPHZ products (Figure 2C, #1 and #2). These products were subsequently purified and analyzed using cyclic voltammetry to assess their redox properties. Consistent with previous studies (Wang et al. 2010; Luo et al. 2020), 1‐OHPHZ exhibits a reversible two‐electron transfer redox reaction, characterized by a pair of symmetrical oxidation and reduction peaks with a midpoint potential of −390.5 mV vs. Ag/AgCl and a peak potential separation of ~28 mV (Figure 2D). By contrast, the ·NO‐reacted 1‐OHPHZ products display more complex redox behavior: each product shows two pairs of oxidation and reduction peaks, with Product 1 showing midpoint potentials of −132 mV and −429 mV, and Product 2 showing midpoint potentials of −70 mV and −462.5 mV vs. Ag/AgCl. These results suggest that they may undergo multistep redox processes or contain more than one redox‐active center (Rafiee et al. 2024). A qualitative assessment of biologically‐mediated reduction of PYO and ·NO‐reacted PYO via incubation with *P. aeruginosa* also suggests that these metabolites retain their redox activity (Figure S2E).

To assess the functional impact of this redox activity, we asked whether these modified phenazines could support anaerobic survival for *P. aeruginosa* . Cells were incubated anaerobically with an oxidizing potential ± phenazine, and cellular survival was monitored over 24 h. In line with previous experiments (Wang et al. 2010), 1‐OHPHZ supports anaerobic survival (Figure 2E). Both ·NO‐transformed phenazines also support anaerobic survival (Figure 2E), which is consistent with their maintenance of redox activity that is a required feature for this phenotype (Glasser et al. 2014). These results demonstrate that ·NO can transform phenazine metabolites and change their chemical properties, without disrupting their usage as electron acceptors for anaerobic survival.

**FIGURE 3:**
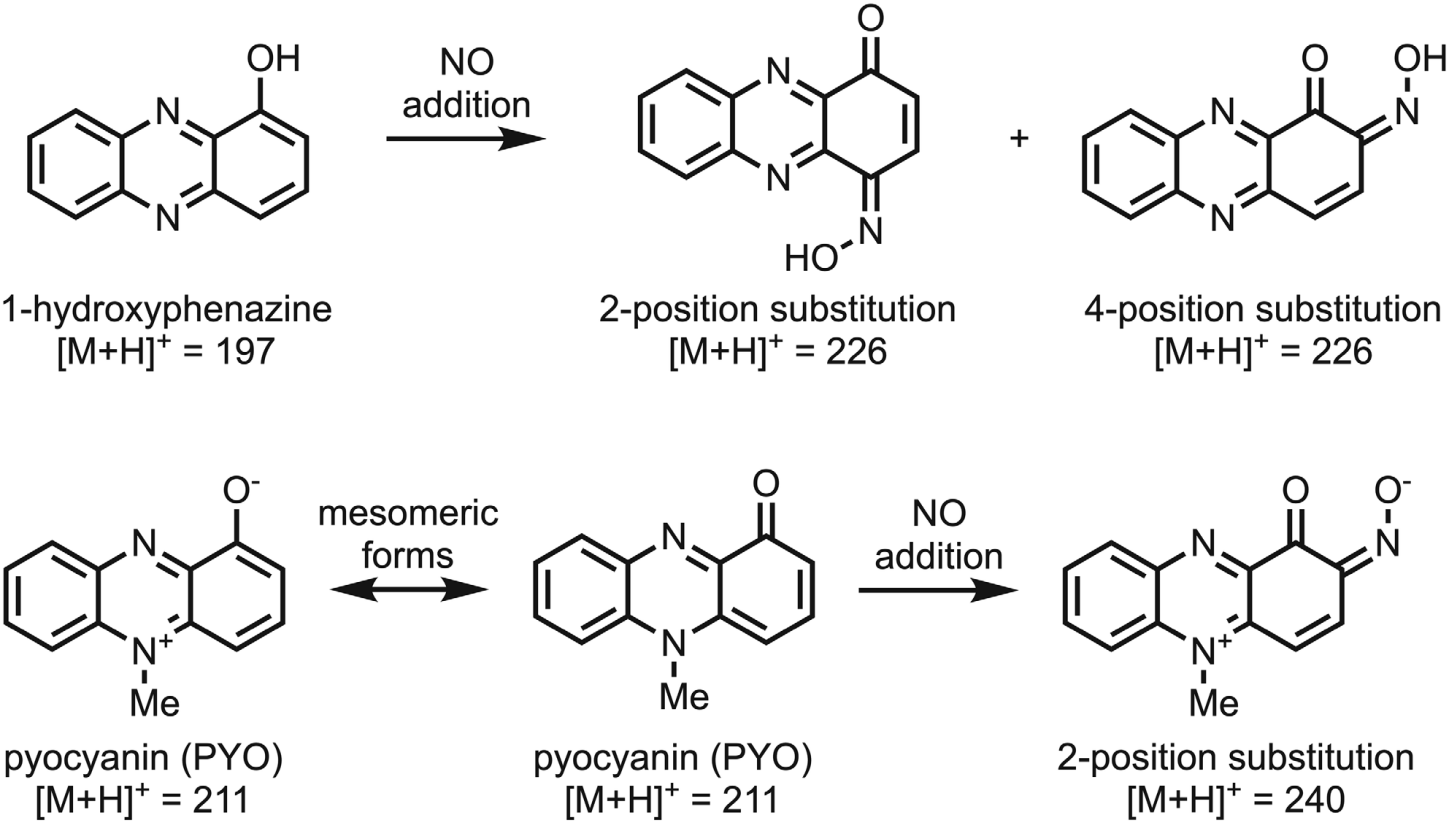
The chemical structures 1‐hydroxyphenazine, pyocyanin, and their nitric oxide adducts, with their detected protonated masses shown.

The mass addition of 29 Da to both PYO and 1‐OHPHZ suggests a conserved reaction mechanism with ·NO (Figure 3). To determine the structure of ·NO‐transformed phenazines, we analyzed the two purified 1‐OHPHZ products using ^1^H nuclear magnetic resonance (NMR) spectroscopy. The resulting spectra reveal the formation of an oxime group on the base pyrazine structure (Figure 3), which is consistent with FTIR results (Figure S3). To elucidate the mechanistic origins of the two possible oxime products, dispersion‐corrected density functional theory calculations were employed using unrestricted dispersion‐corrected DFT (ωB97X‐D/set cc‐pvDZ‐CPCM (H_2_O); see Data S1 and Figures S6–S12, Table S2). ·NO is recognized as a stable free radical, reacting rapidly with redox‐active metals and other free radicals, yet relatively unreactive in processes involving two‐electron oxidations or reductions (Ford and Miranda 2020). However, the ability of ·NO to form N_2_O_2_, a weak electrophilic dimer, in the presence of aromatic hosts, lends itself to nucleophilic attack via the more negatively charged sites on 1‐OHPHZ (Zhao et al. 2005).

**FIGURE 4:**
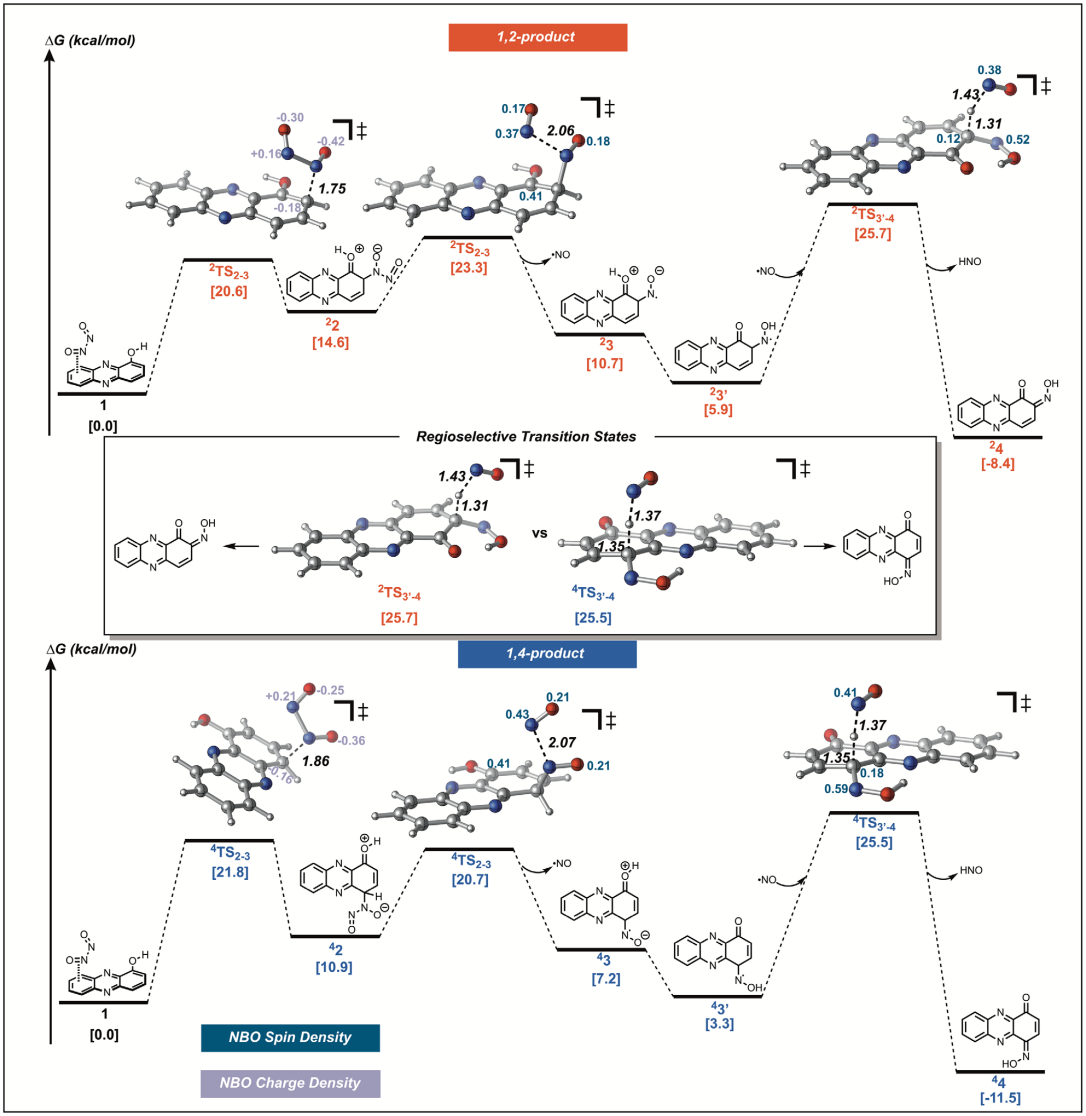
Proposed structures and reaction mechanisms of phenazine‐NO interactions. Free energy pathway for the 1,2‐ and 1,4‐addition of ·NO to 1‐hydroxyphenazine. Free energies (in kcal/mol) were calculated at the uωb97xD/6–311 + g(d,p)‐CPCM(H_2_O)//uωb97xD/cc‐pvDZ‐cpcm(H_2_O) level of theory. Bond lengths are presented in angstroms, written in black.

In our case, N_2_O_2_ is introduced as the reactive partner by coordination with 1‐OHPHZ, approximately 3.10 Å above the aromatic rings (Figure 4). From this complex, we found that N_2_O_2_ then undergoes a nucleophilic attack (via a barrier of ~21 kcal/mol) at either the two or four position due to their increased nucleophilicity (Data S1), leading to the corresponding NONO adducts. In turn, these adducts can undergo N‐N homolytic cleavage to form ·NO and an open‐shell hydroxyamino‐like intermediate (^2^3 and ^4^3) that can rapidly tautomerize (Data S1) to the lowest‐energy N–OH adducts (Harcourt 1990). Finally, both pathways undergo hydrogen atom transfer (HAT) via an energetically feasible barrier (~26 kcal/mol), yielding the desired products.

Charge density analysis was key to determining the initial step of the reaction mechanism. Natural bond orbital (NBO) analysis was used to visualize the charge density at each atomic position. Initially an aza‐Michael Addition type mechanism was postulated, but NBO calculations revealed that the 2‐ and 4‐positions are electron rich. The charge densities for the N_2_O_2_ addition are shown in Figure 4, demonstrating the respective nucleophilicity and electrophilicity of each reactant. NBO spin density analysis was also used to confirm the open‐shell nature of the homolytic cleavage and HAT transition states. The radical character is delocalized throughout the phenazine ring as seen by the selected spin densities (Figure 4). Due to the irreversibility of the HAT transition state, this was determined to be the regioselective step that establishes the structurally distinct products. All steps leading up to the HAT can be considered reversible, and due to a difference of only 0.2 kcal/mol in the ΔG^‡^ of the regioselective transition states, a mixture of products is expected.

Taken together, these results show that ·NO chemically transforms some phenazine metabolites into two structurally distinct molecules via a mechanism that employs nucleophilic attack of the aromatic ring on the ·NO dimer, homolytic cleavage, and hydrogen atom transfer. While these nitrosylated phenazines maintain redox properties that can support *P. aeruginosa* anaerobic survival once they are formed, why the presence of PYO and ·NO negatively impacted *P. aeruginosa* during growth remained a puzzle that we next sought to address.

### 2.3 PYO‐NO Reactivity Is Acutely Toxic

**FIGURE 5:**
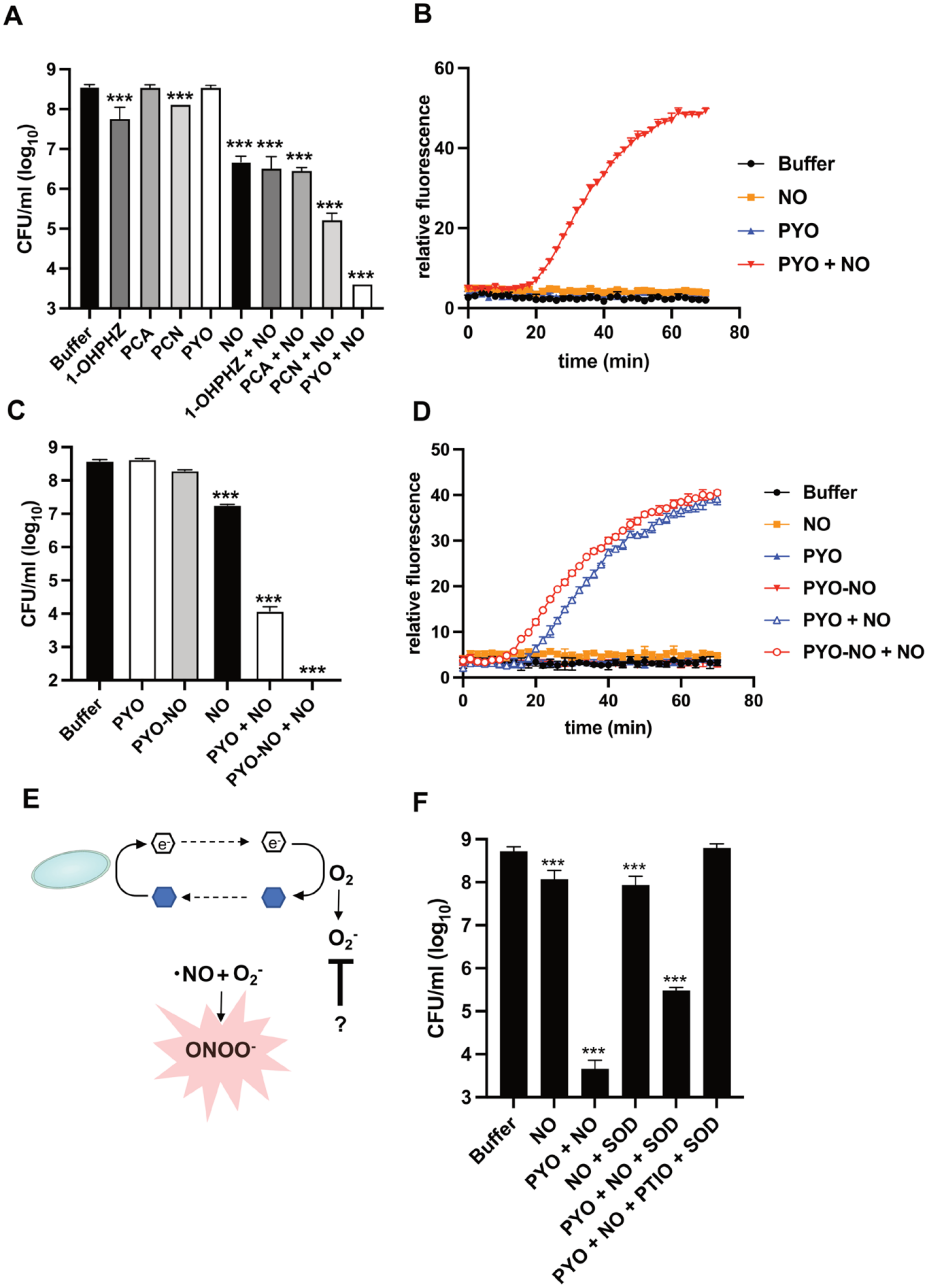
Pyocyanin‐nitric oxide reactivity is acutely toxic to *P. aeruginosa* . (A) Bacterial survival reported as CFU/mL of *P. aeruginosa* after incubation with 100 μM 1‐OHPHZ, phenazine‐1‐carboxylic acid (PCA), phenazine‐1‐carboxamide (PCN), or PYO ±1.5 mM DEA‐NONOate (‘·NO’); *n* = 3 biological replicates, mean ± SD. (B) Representative propidium iodide (PI) fluorescence of *P. aeruginosa* during incubation with 100 μM PYO, 1.5 mM DEA‐NONOate (‘·NO’) or PYO + ·NO. (C) Bacterial survival reported as CFU/mL of *P. aeruginosa* after incubation with 100 μM PYO, 1.5 mM DEA‐NONOate (‘·NO’), PYO + NO or PYO‐NO (product) + ·NO for 1 h; *n* = 3 biological replicates, mean ± SD. (D) Representative PI fluorescence of *P. aeruginosa* during incubation with 100 μM PYO, 1.5 mM DEA‐NONOate (‘·NO’), PYO + ·NO, or PYO‐NO + ·NO. (E) Proposed scheme for how PYO and ·NO may integrate with phenazine redox cycling and oxygen to produce the reactive nitrogen species ONOO^−^. (F) Bacterial survival reported as CFU/mL of *P. aeruginosa* after incubation with 100 μM PYO ±1.5 mM DEA‐NONOate (‘·NO’) ± recombinant superoxide dismutase (SOD; ~3000 U/mL) ±·NO scavenger carboxy PTIO (PTIO; 1.5 mM) for 1 h; *n* = 3 biological replicates, mean ± SD. ***p < 0.001, one‐way ANOVA with Tukey multiple comparisons vs. buffer.

To gain insight into why the reaction between phenazines and ·NO is toxic to *P. aeruginosa* , we exposed *P. aeruginosa* to either ·NO, phenazines, or a combination of both for various periods to assess how their interactions affect cell viability. When *P. aeruginosa* is exposed acutely to high concentrations of ·NO for 1 h, it loses viability (Figure 5A). The ·NO detoxifying enzyme Fhp does not contribute to this survival (Figure S4A), potentially due to lack of induction in these conditions. When it is exposed to ·NO alongside 1‐OHPHZ or phenazine 1‐carboxylic acid (PCA), there is a minor combined loss in viability (Figure 5A). Co‐exposure to ·NO and phenazine 1‐carboxamide (PCN) results in more pronounced death (Figure 5A). However, the combined toxicity for each of these phenazines is relatively minor compared to that effected by PYO and ·NO (Figure 5A), where ~1‐million‐fold loss in viability is observed. Further, Fhp also does not contribute to this survival (Figure S4A). To understand the kinetics of PYO‐NO combined toxicity, cells were treated with propidium iodide (PI) during exposure to the molecules. PI is occluded from sufficiently polarized cellular envelopes but can diffuse across compromised envelopes, indicating these cells have low metabolic activity and/or are dead (Shi et al. 2007; Spero and Newman 2018). When cells are treated with either PYO or ·NO, there is no change in the PI relative fluorescence, which is similar to background (Figure 5B). However, when cells are incubated with PYO and ·NO simultaneously, there is a significant increase in PI relative fluorescence (Figure 5B). These results indicate that the PYO reaction with ·NO causes rapid membrane depolarization, promoting PI cellular influx and suggesting a mechanism for cell death.

We wondered whether the product formed when PYO and ·NO react was causing the toxicity, or if the toxicity stemmed from how PYO reacts with ·NO. To distinguish between these possibilities, we exposed cells to ·NO, PYO, or the ·NO‐derivative PYO‐NO singly and in combination with ·NO and monitored viability. Incubation with each phenazine on its own did not significantly decrease cell viability (Figure 5C). Incubation with ·NO alone decreased viability ~1 log_10_ (Figure 5C). Incubation with PYO and ·NO simultaneously decreased viability by ~4.5 log_10_. Surprisingly, incubation of previously ·NO‐reacted PYO (PYO‐NO) with ·NO for a second time resulted in > 8 log_10_ loss in viability, with viability being below the limit of detection of 100 cells/mL (Figure 5C). These findings are consistent with the rapid increase in PI staining associated with PYO‐NO reactivity with ·NO (Figure 5D). These results demonstrate that PYO‐NO itself does not cause acute toxicity but that the toxicity derives from its formation and/or from subsequent reactions between PYO‐NO and ·NO. The rapid depolarization of the bacterial cell envelope during these reactions rationalizes its efficient killing.

An important feature of phenazines produced by *P. aeruginosa* is their redox cycling capacity, which promotes *P. aeruginosa* anaerobic survival (Wang et al. 2010). While such recycling can be achieved under strictly anoxic conditions using electrodes to regenerate oxidized phenazines, under (hyp)oxic conditions, reduced phenazines can be re‐oxidized by molecular oxygen (O_2_) (Figure 5E). Yet phenazine oxidation by O_2_ produces superoxide radicals (Nishikimi et al. 1972), which can then react with ·NO to produce peroxynitrite, one of the strongest nitrosative stress agents (Szabo et al. 2007) (Figure 5E). Accordingly, we hypothesized that superoxide production may drive the rapid toxicity that occurs when PYO reacts with ·NO and reasoned that preventing superoxide formation might curtail this toxicity (Figure 5E). To test this hypothesis, we incubated cells with a combination of PYO and ·NO plus recombinant, exogenous superoxide dismutase (SOD), which catalyzes the dismutation of superoxide to hydrogen peroxide, thereby lowering superoxide concentration (Nozik‐Grayck et al. 2005). Cells were incubated for 1 h with the various treatments and viability was determined. The addition of SOD had no effect on ·NO toxicity alone, but it did partially rescue the toxicity of PYO and ·NO provided simultaneously (Figure 5F). Addition of the ·NO scavenger carboxy‐PTIO (PTIO) completely alleviates ·NO toxicity as well as PYO and ·NO combined toxicity (Figure 5F). However, ·NO scavenging must occur at the beginning of the incubation, since addition of PTIO after initial incubation with PYO and ·NO does not rescue *P. aeruginosa* viability (Figure S4B). Together, these data demonstrate that ·NO can react with phenazines and impact cell viability depending on the ·NO‐molecule pairing; further, the production of superoxide via redox cycling under oxic conditions contributes to this toxicity.

### 2.4 Nitric Oxide‐Enhanced PYO Killing Is Species‐Specific and Cell‐Intrinsic

**FIGURE 6:**
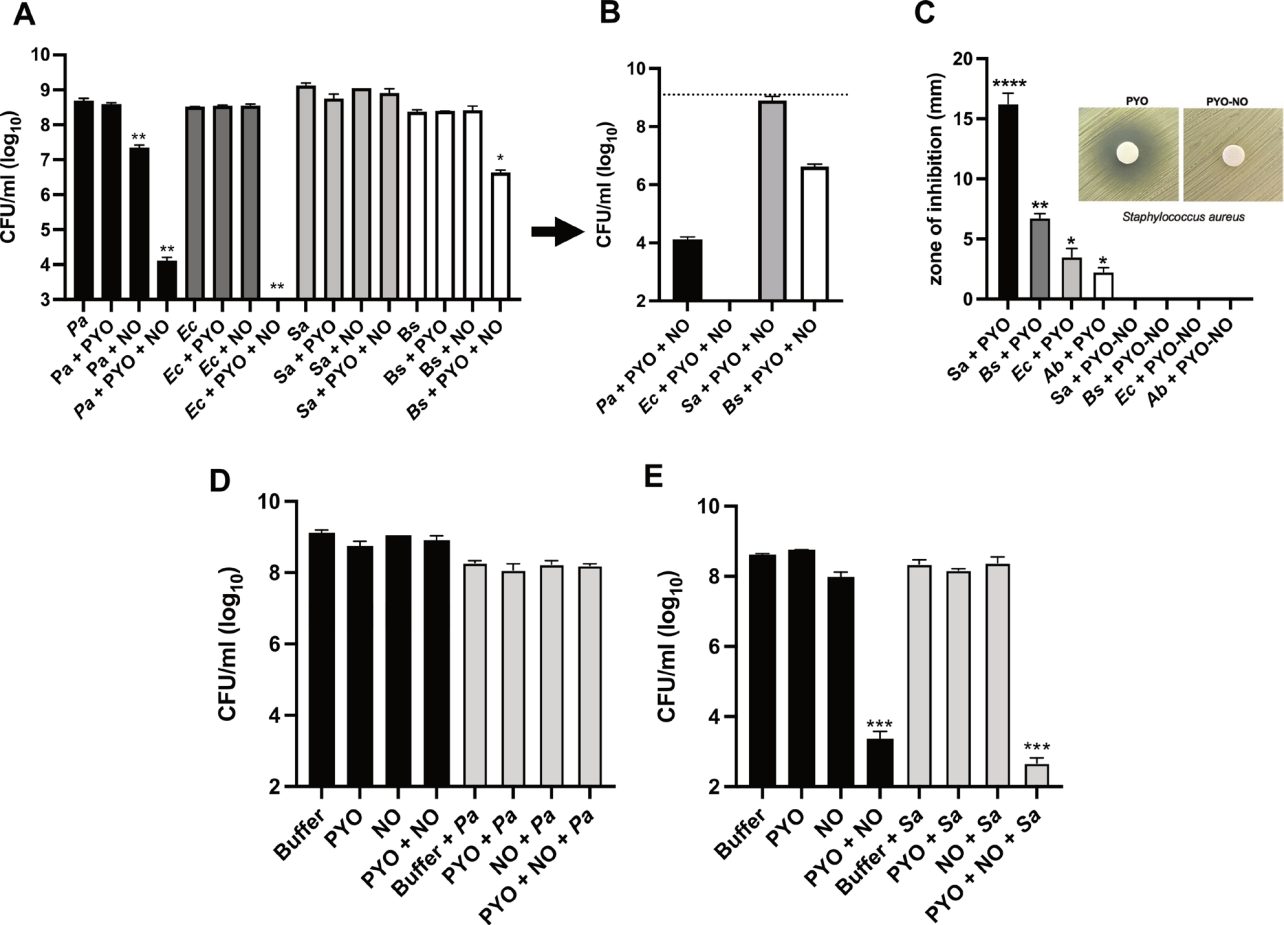
Pyocyanin‐nitric oxide toxicity has species specificity with cell‐intrinsic resistances. (A) Bacterial survival reported as CFU/mL of *P. aeruginosa, Escherichia coli*, *Staphylococcus aureus* , and *Bacillus subtilis* after incubation with 100 μM PYO, 1.5 mM DEA‐NONOate (‘·NO’) or PYO + NO for 1 h, with (B) inset to the right showing PYO + NO killing for each species; *n* = 3 biological replicates, mean ± SD. *p < 0.05, ***p < 0.001, one‐way ANOVA with Tukey multiple comparison vs. strain‐only control. (C) Radial zone of inhibition measurements for *B. subtilis* , *E. coli* , and *A. baumannii* after disk exposure for 24 h to PYO or PYO‐NO gradients, with *S. aureus* insert; *n* = 3 biological replicates, mean ± SD. *p < 0.05, **p < 0.01, ****p < 0.0001, unpaired *t*‐test PYO vs. PYO‐NO. (D) Bacterial survival reported as CFU/mL of *S. aureus* ± incubation with *P. aeruginosa* ± 100 μM PYO, ±1.5 mM DEA‐NONOate (‘·NO’); *n* = 3 biological replicates, mean ± SD. (E) Bacterial survival reported as CFU/mL of *P. aeruginosa* ± incubation with *S. aureus* ± 100 μM PYO, ±1.5 mM DEA‐NONOate (‘·NO’); *n* = 3 biological replicates, mean ± SD. ***p < 0.001, one‐way ANOVA with Tukey multiple comparisons vs. buffer.

Having established the likely mechanism whereby nitrosylation of PYO kills *P. aeruginosa* , we were curious whether other cell types were similarly sensitive. Strains of *Escherichia coli* , *Staphylococcus aureus* , and *Bacillus subtilis* were exposed to ·NO and/or PYO for 1 h before dilution on solid medium. While PYO is well‐known to possess antimicrobial activity against many organisms (Baron and Rowe 1981), the 1‐h incubation with PYO did not significantly change viability of *E. coli* , *S. aureus* , or *B. subtilis* (Figure 6A). When compared to *P. aeruginosa* , *E. coli* was more sensitive to PYO and ·NO cotreatment (Figure 6A). By contrast, both *S. aureus* and *B. subtilis* were more resistant to PYO and ·NO cotreatment compared to *P. aeruginosa* , with *S. aureus* being completely resistant (Figure 6A). The resistance of *S. aureus* to PYO and ·NO cotreatment was particularly interesting, since PYO has known antimicrobial effects against *S. aureus* (Baron and Rowe 1981). To determine if the transformation of PYO by ·NO altered its antimicrobial activity, *S. aureus* was placed on a solid medium with a saturated disk of PYO or PYO‐NO, and growth inhibition in the vicinity of the disk was measured. Consistent with previous results (Baron and Rowe 1981; Noto et al. 2017), PYO inhibits *S. aureus* growth, but PYO‐NO does not (Figure 6C). *B. subtilis* , *Acinetobacter baumannii* , and *E. coli* were also completely unphased by PYO‐NO (Figure 6C). Because *S. aureus* frequently competes with *P. aeruginosa* for infectious niches, we wondered whether its resistance to PYO‐NO might give it an advantage over *P. aeruginosa* in co‐culture. To put this to the test, we compared the sensitivity of *S. aureus* and *P. aeruginosa* to PYO, ·NO, and the combination in mono or mixed cultures. While *P. aeruginosa* typically outcompetes *S. aureus* over time (Hotterbeekx et al. 2017; Alford et al. 2022), this 1‐h incubation did not change the viability of *S. aureus* or *P. aeruginosa* (Figure 6D). Additionally, the exposure time to PYO was insufficient to decrease the viability of *S. aureus* (Figure 6D). By contrast, when *P. aeruginosa* was exposed to PYO and ·NO simultaneously, there is a 5log_10_ drop in viability, regardless of whether *S. aureus* is present (Figure 6E). Yet *S. aureus* remained completely resistant to PYO and ·NO simultaneous exposure (Figure 6D). These results illustrate that *S. aureus* possesses intrinsic resistance to PYO and ·NO reactivity. Collectively, these results demonstrate that PYO and ·NO toxicity is species‐specific.

## 3 Discussion

NO is commonly assumed to be broadly reactive, but specific molecular features react preferentially in ways that have important biological consequences. For example, the phenolic ring of tyrosine and the thiol side chain of cysteine are both reactive with ·NO and its derivatives, thereby altering protein function (Hess and Stamler 2012; Radi 2013). The main contribution of this work is to expand this principle from protein biochemistry into the secreted small molecule landscape. By using nitrosylation of phenazine metabolites as a model system, we demonstrated that certain phenazine metabolites possessing 1‐hydroxy functional groups are ·NO‐reactive, and that this reactivity transforms the properties of the phenazines in a manner that alters their biological effects.

How does this change in impact manifest? Our results indicate that the change in reactivity of the nitrosylated phenazine and/or the formation of the adduct itself is responsible for its altered biological effects, but varies according to the specific phenazine and bacterial species in question. Specifically, we found that when PYO reacts with ·NO, it induces rapid loss of *P. aeruginosa* viability, but this loss does not occur when the structurally similar phenazine 1‐OHPHZ reacts with ·NO. At least part of this toxicity likely results from superoxide production that could be generated during phenazine redox cycling (Lau et al. 2004; Hassan and Fridovich 1980; Hassett et al. 1992). Important differences between PYO and 1‐OHPHZ are their relative reduction potential and PYO's more rapid redox cycling compared to 1‐OHPHZ, which could partly explain the differences between the two phenazines to synergize with ·NO to impart toxicity (Wang et al. 2010). Because addition of SOD did not fully rescue this toxicity, multiple effects likely contribute to the phenotype, including the ability of ·NO to rapidly convert into other toxic N‐oxides (Wink and Mitchell 1998). Additionally, we observed that nitrosylated PYO maintains reactivity with ·NO that causes rapid loss in viability. While we did not observe multiple ·NO additions to PYO in our analyses, we did observe a mass addition of 54 in certain circumstances (Figure S2A) that might represent NO_2_ addition to the phenazine scaffold; therefore, additional reaction mechanisms between PYO and ·NO remain to be explored.

·NO‐mediated toxicity in the presence of PYO also depends on the species that is in proximity to the metabolite‐NO reaction. For instance, the Gram‐positive pathogen *S. aureus* is intrinsically resistant to PYO reacting with ·NO and is also insensitive to the PYO‐NO adduct. While the specific resistance mechanism awaits future determination, previous studies indicate *S. aureus* resists PYO and ·NO stress in part by using a lactic acid fermentative metabolism and inducing stress response and ·NO detoxification pathways (Gusarov et al. 2009; Noto et al. 2017; Richardson et al. 2008; Vitko et al. 2015).

Both PYO and the structurally similar phenazine 1‐OHPHZ form new products when nitrosylated. Our structural analyses of 1‐OHPHZ reacted with ·NO identified both a 1,2‐addition and 1,4‐addition to the ring, while PYO reacted with ·NO resulting in only one product with the correct mass for ·NO addition. Considering the structural similarities between PYO and 1‐OHPHZ, the 1‐hydroxy functional group is likely the conserved structural feature driving ·NO reactivity for both molecules, with the pyrazine methyl group likely preventing 1,4‐addition to PYO. This reaction mechanism is consistent with defined mechanisms for phenolic scaffolds to react with ·NO and other related N‐oxides (Janzen et al. 1993). While we were able to detect PYO‐NO reaction products in both oxic and anoxic environments, we found that the addition of PYO and ·NO simultaneously to growing cultures of *P. aeruginosa* only results in cell death once the cultures reach stationary phase. An important change that occurs as *P. aeruginosa* transitions into this phase is increased hypoxia due to rapid oxygen consumption in a turbid culture, which can result in the chemical reduction of phenazines even when the air‐liquid interface is oxic (Price‐Whelan et al. 2007). We thus infer that reduced PYO is preferentially reactive with ·NO and drives the rapid loss of viability over short time periods.

Phenazines are bacterial‐derived secreted metabolites that are widespread in nature and disease, where they can support anaerobic metabolism and exert antimicrobial activity, among other functions (Wang et al. 2010; Thomashow and Weller 1988; Baron and Rowe 1981; Mazzola et al. 1992; Vilaplana and Marco 2020). Phenazines can also accumulate in biologically important contexts; for example, direct measurements of phenazines from human lungs indicate they can reach concentrations of ~100 μM, which served as the basis for our in vitro experimentation to examine the effects of PYO and ·NO reactivity on bacteria (Wilson et al. 1988). The environments in which phenazines are made are also replete with ·NO, which can be biotically or abiotically derived (Bisson, and University of Reading, Department of Soil 1994; Zafiriou et al. 1980; Kampschreur et al. 2007; Wang et al. 2024). While directly measuring ·NO concentrations in vivo is challenging due to the molecule's rapid diffusion and short half‐life (Andrabi et al. 2023), estimates of activated macrophage‐derived ·NO concentrations are generally sustained in the micromolar range over extended periods of time (Lewis et al. 1995; Zhuang and Wogan 1997). Further, measurements of stable oxidation products of ·NO range from tens to hundreds of micromolar concentrations (Wilson et al. 1988; Lewis et al. 1995; Utaisincharoen et al. 2000; Fujii and Osaki 2023; Palmieri et al. 2020); our delivery of ·NO from small molecular donors captures these ranges (Figure S5) (Wang et al. 2024). The widespread occurrence of ·NO in small molecule‐dominated ecosystems suggests that ·NO‐mediated small molecule transformation may be an important and overlooked chemical process. Consistent with this prediction, a recent analysis of wastewater identified an ·NO‐modified version of ciprofloxacin, which is a synthetic antibiotic with important clinical relevance (Brienza et al. 2020). Given the importance of both secreted metabolites and ·NO for both human and microbial physiology, our findings suggest that these ·NO‐mediated transformations may have important implications for human health and the environment.

A major challenge in understanding small molecule function is dissecting their roles in complex ecosystems. This problem is compounded by our limited capacity to identify unknown metabolites from chemically complex environments without a priori knowledge (Giera et al. 2022). Our analysis of ·NO‐transformed phenazines illustrates that specific mass and polarity shifts occur following ·NO‐phenazine reactivity, which suggests these chemical changes could be used to identify other molecules undergoing ·NO‐mediated transformations. Here we have taken a reductionist approach to identify chemical parameters that facilitate ·NO reactivity with phenazines and optimized analytic methods to detect them while defining their biological importance; we hope to apply this logic to diverse and dynamic ecosystems with the goal of uncovering the breadth of metabolite chemical transformations facilitated by ·NO and their consequences.

## 4 Materials and Methods

### 4.1 Bacterial Strains and Reagents

Experiments were performed using *Pseudomonas aeruginosa* strain UCBPP‐PA14 (*Pa*) unless otherwise noted. Experiments involving liquid culture were performed in lysogeny broth (LB) at 37°C with aeration at 250 rpm (Innova) unless otherwise stated. Nitric oxide donors DETA‐NONOate (Cayman #82120) and DEA‐NONOate (#82100) were acquired from Cayman Chemicals. DETA‐NONOate has a half‐life of 20 h at 37°C and 56 h at 22°C–25°C (pH 7.4) and liberates 2 mol of ·NO per mole of parent compound (Wang et al. 2024; Hrabie et al. 1993; Keefer et al. 1996). DEA‐NONOate has a half‐life of 2 min at 37°C and 16 min at 22°C–25°C (pH 7.4) and liberates 1.5 mol of ·NO per mole of parent compound (Keefer et al. 1996; Maragos et al. 1991); see Figure S5A for ·NO release kinetics. Other strains utilized were *Staphylococcus aureus* USA300 LAC, *Acinetobacter baumannii* ATCC 17978, *Escherichia coli* strain MG1655, *Bacillus subtilis* strain 168, all from the Newman lab strain collection.

### 4.2 Growth in Nitric Oxide

Growth in the presence of nitric oxide was performed in either 5 mL volumes in 15 mL culture tubes or in 96‐well plates, and in both cases ·NO was provided via DETA‐NONOate. Bacterial strains were grown overnight in LB, and the following day cultures were diluted to OD_500_ of 0.05 in fresh media. DETA‐NONOate stocks (500 mM in 10 mM NaOH) were thawed from −80°C and diluted to indicated concentrations in LB before pipetting 150 μL into a 96‐well flat bottom plate or mixing with 5 mL LB in a 15 mL culture tube. For PYO supplementation experiments, PYO was diluted into LB at 100 μM. Diluted bacteria were inoculated 1:30 into the 96‐well plate or into a 15 mL culture tube. 96‐well plates were placed in a Biotek Epoch2 plate reader at 37°C with continuous orbital shaking and OD_500_ absorbance readings captured hourly, and 15 mL culture tubes were placed into a 37°C incubator with 250 rpm shaking, and samples were taken over time for absorbance readings.

### 4.3 Thin Layer Chromatography

Phenazines were spotted on analytical silica TLC plates (vendor) using 5 mL capillaries and allowed to dry. Dried plates were placed in a small vial with the following mobile phases: PYO and derivatives utilized a mobile phase of 50:50 methanol:chloroform, while 1‐OHPHZ and derivatives utilized a mobile phase of 5:95 methanol:chloroform. Samples were run until the solvent front reached near the end of the plate and marked, with Rf values calculated as a fraction a product migrated as a function of the solvent front.

### 4.4 Disk Diffusion Assay

Overnight bacterial cultures were streaked onto LB agar using a sterile cotton‐tipped applicator. Sterile paper disks were placed onto the plate and spotted with 10 μl of 2 mM compound or solvent control. Plates were placed at 37°C incubator overnight, and radial zones of inhibition were measured.

### 4.5 Pyocyanin Purification

Pyocyanin (PYO) was synthesized and purified as previously described (Costa et al. 2017). Briefly, ~500 mg of phenazine methosulfate (Sigma, CAS #299‐11‐6) was dissolved in 500 mL 20 mM ammonium bicarbonate and placed in a fluorescent light box overnight at room temperature and constant stirring. The following day, an aqueous extraction was performed 6× with 100 mL washes of dichloromethane (DCM; Baker) in a separatory funnel and fractions collected and pooled. DCM extract was then acidified with 125 mL 0.1 M HCl, and the aqueous phase was extracted twice. Extracted phases were mixed with 2.5 mL 10 M NaOH and extracted 6× with 100 mL DCM and fractions collected and pooled. DCM was removed via Rotovap, and the resulting solid was resolubilized in ~5 mL methanol:DCM (20:80). Samples were dried overnight under N_2_ gas stream. The following day, the sample was dissolved in 2 mL DCM and washed with 30 mL *n*‐hexanes. Material was collected on vacuum filter and transferred to permanent storage vial. Purity was assessed using LC–MS.

### 4.6 Phenazine Reactivity and Extraction

For nitric oxide reactivity with pyocyanin (PYO) and 1‐hydroxyphenazine (1‐OHPHZ), purified PYO or commercially‐available 1‐OHPHZ (Fisher; CAS #528‐71‐2) was resuspended in 20 mM HCl. For bulk synthesis and purification, phenazines were either diluted to 500 μM (1‐OHPHZ) in HCl and sparged with N_2_ gas for 10 min, or diluted to 2 mM (PYO) with deoxygenated 20 mM HCl or 1× PBS within an anaerobic chamber (Coy Laboratory Products). For oxic conditions, phenazines were diluted to 500 μM in ambient air and ambient air‐exposed buffers. For sparged samples, phenazines were exposed for ·NO gas (Mesagas; ID 1660) with a relative flow rate of 10 mL/min for up to 2 h. For samples in the anaerobic chamber, DEA‐NONOate (500 mM stock in 10 mM NaOH) was added to a final donor concentration of 3 mM and reaction proceeded overnight. PYO nitroso products (PYO‐NO) were extracted via salting out with MgSO_4_ (1 g) and 1 mL acetonitrile (ACN; Fisher). ACN fractions were separated via preparative thin layer chromatography (TLC) (Sigma) and separated with a mobile phase of 1:1 methanol:chloroform (Sigma). For 1‐OHPHZ, nitroso products (1‐OHPHZNO) precipitated following reactivity and were resolubilized in methanol prior to separation via preparative TLC and a mobile phase of 5:95 methanol:chloroform. In both cases, desired products were eluted with methanol from silica via flash chromatography. Fractions were collected and dried under N_2_ stream, and purity was assessed using LC–MS.

### 4.7 Cyclic Voltammetry

Cyclic voltammetry (CV) experiments were performed using a CH Instruments potentiostat (CHI760E) at a scan rate of 20 mV/s. The electrochemical cell consisted of a 3‐mm glassy carbon working electrode (BASi), an Ag/AgCl (3 M KCl) reference electrode (BASi), and a platinum wire counter electrode. Phenazines and phenazine‐NO derivatives were prepared as 10 mM stock solutions in methanol and subsequently diluted 100‐fold to a final concentration of 100 μM in 1× phosphate‐buffered saline (PBS, pH 7.3). All solutions were purged with N_2_ for at least 10 min prior to measurement, and experiments were conducted under a continuous N_2_ flow. Before each run, the glassy carbon electrode was polished using 0.05 μm gamma alumina powder.

### 4.8 Anaerobic Survival Assay

Anaerobic survival experiments were performed using a 96‐potentiostat system as previously described (Ciemniecki et al. 2024). Cells were cultured for 20 h in a *Pseudomonas* MOPS minimal medium (100 mM MOPS pH 7.2, 43 mM NaCl, 93 mM NH_4_Cl, 3.7 mM KH_2_PO_4_, 1 mM MgSO_4_), pelleted for 5 min at 8000 × *g*, washed twice in minimal medium, resuspended at OD_500_ of 75, and transferred to the anaerobic chamber with an ambient temperature of 33°C and ~2% H_2_. Bacteria were diluted to an OD of 15 with N_2_‐sparged media, and 10 μL of bacteria were placed into a 96‐well electrochemical plate containing 190 μL of media and 100 μM 1‐OHPHZ or its derivative phenazines. The plate contained a carbon working electrode and counter electrode, plus a Ag/AgCl reference electrode. Plates were covered with a slit‐seal cover (BioChromato #R80.120.00) and aluminum cover (Diversified Biochem #ALUM‐1000). Plates were incubated statically at 33°C in a 96‐potentiostat system, with each well held at 0 mV vs. Ag/AgCl reference electrode. Cyclic voltammograms and current readouts were collected over 24 h, at which point 10 μL of cells was removed for CFU plating. All plastics for use within the anaerobic chamber were present for at least 3 days before use to allow sufficient degassing.

### 4.9 Structural Analyses

#### 4.9.1 Infrared Spectroscopy

Purified phenazines were analyzed via Fourier Transform Infrared (FTIR) spectroscopy on a Nicolet 6700 spectrometer. Solid samples were loaded onto the spectrometer fitted with a diamond‐tipped ATR adaptor under a continuous N_2_ stream at a flow rate of 40ft^3^/h. Spectra were captured for 64 scans with Resolution 4. A corrected interferogram was obtained by subtracting a scan background blank, and spectral values were exported from the OMNIC software subsequent to plotting.

#### 4.9.2 Nuclear Magnetic Resonance

##### 4.9.2.1 Compound Preparation

Phenazines were reacted and extracted in bulk as described in the Section 4.6. Approximately 5 mg of product was solubilized with deuterated methanol and loaded into 5 mm, 7 in. NMR tubes (Norell).

##### 4.9.2.2 Chemical Methods

^1^H NMR spectra were recorded on a Varian Inova spectrometer (500 MHz), in the stated solvents as a reference for the internal deuterium lock. The chemical shift data for each signal are given as δ_H_ in units of parts per million (ppm) relative to tetramethylsilane (TMS) where δ_H_ (TMS) = 0.00 ppm. The spectra are calibrated using the solvent peak with the data provided by Fulmer et al. (2010) The multiplicity of each signal is indicated by s (singlet); br s (broad singlet); d (doublet); dd (doublet of doublets), ddd (doublet of doublet of doublets), t (triplet), q (quartet), dq (double of quartet) or m (multiplet). The number of protons (*n*) for a given resonance signal is indicated by nH. Where appropriate, coupling constants (*J*) are quoted in Hz and are recorded to the nearest 0.1 Hz. Identical proton coupling constants (*J*) are averaged in each spectrum and reported to the nearest 0.1 Hz. The coupling constants were determined by analysis using MestReNova version 14.3.0 software. Infrared (IR) spectra were obtained from neat samples, either as liquids or solids using a diamond ATR module. The spectra were recorded on a Nicolet 6700 spectrometer. Absorption maxima are reported in wavenumbers (cm^−1^).

##### 4.9.2.3 2‐(Hydroxyimino)Phenazin‐1(2H)‐One

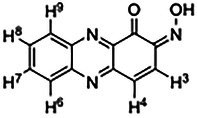

𝝂¯_max_ (solid)/cm^−1^ 3309, 2925, 2854, 1742, 1608, 1509, 1251, 1177, 1037, 829; ^1^H NMR (500 MHz; MeOD) δ_H_ 8.33 (2H, d, *J* 8.2, H^7^ & H^8^), 8.28 (1H, d, *J* 10.4, H^4^), 8.02 (1H, dd, *J* 8.2, 7.7, H^9^), 7.95 (1H, dd, *J* 8.2, 7.7, H^6^), 6.78 (1H, d, *J* 10.4, H^3^); LRMS *m/z* (ESI^+^) 226.2 ([M + H]^+^ 100%).

##### 4.9.2.4 4‐(Hydroxyimino)Phenazin‐1(4H)‐One

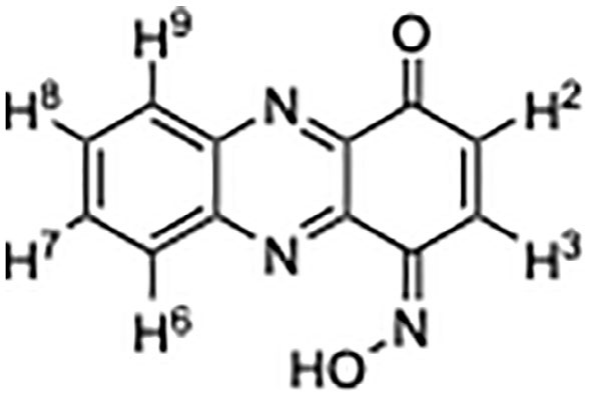

𝝂¯_max_ (solid)/cm^−1^ 3309, 2925, 2854, 1742, 1608, 1509, 1251, 1177, 1037, 829; ^1^H NMR (500 MHz; MeOD) δ_H_ 8.38 (2H, d, *J* 8.6, H^6^), 8.14 (1H, d, *J* 8.2, H^9^), 7.98 (1H, ddd, *J* 8.2, 7.7, H^7^), 7.91 (1H, dd, *J* 8.0, 7.7, H^8^), 7.65 (1H, d, *J* 10.2, H^2^), 6.85 (1H, d, *J* 10.2, H^3^); LRMS *m/z* (ESI^+^) 226.2 ([M + H]^+^ 100%).

The above represents a doublet appearing at 4.56 ppm on the corrected (to the residual non‐deuterated solvent) *x* axis. This peak integrates to two protons and has a coupling constant (also referred to as *J* value) of 7.23 Hz.

### 4.10 Phenazine‐Nitric Oxide Toxicity

Overnight bacterial cultures were pelleted for 3 min at 8000 × *g* at room temperature and resuspended at an OD_500_ of 1 for *Pa* in PBS. For anaerobic experiments, pellets were taken to the anaerobic chamber and resuspended in de‐gassed PBS. Cells were diluted to an OD_500_ of 1 for *Pa*, or ~10^8^ colony forming units (CFU) per mL for other bacterial species. Cells were incubated for 1 h with 1.5 mM DEA‐NONOate (or indicated concentration) ±100 μM phenazine at room temperature and serial‐diluted on LB agar for CFU enumeration. For superoxide dismutase (SOD; Sigma # S5395‐15KU) addition, SOD was added at ~3000 U per mL. For carboxy PTIO addition, PTIO (Sigma #C22‐10MG) was added at a final concentration of 1.5 mM. For bacterial interaction experiments, equal ratios of *P. aeruginosa* and *S. aureus* were mixed with a final OD_500_ of 1 and incubated with DEA NONOate ± phenazine, and surviving cells were recovered by selective plating on mannitol salt agar to select for *S. aureus* or *Pseudomonas* isolation agar to select for *P. aeruginosa* .

### 4.11 Propidium Iodide Spectroscopy

Overnight bacterial cultures were pelleted for 3 min at 8000 × *g* and resuspended in an equal volume of 1× PBS. OD_500_ values were obtained and were normalized to OD_500_ = 1 in PBS. DEA‐NONOate was provided as the ·NO donor at a final concentration of 1.5 mM, and PYO or PYO‐NO was supplemented at a final concentration of 100 μM. Propidium iodide (PI) was added at a final concentration of 66.8 μM. After combining all reagents with cells, 100 μL of cell suspensions were placed in black‐sided 96‐well plates and loaded onto a SpectraMax M5 multi‐mode microplate reader. PI fluorescence was monitored every minute (ex: 535, em: 620).

### 4.12 LC–MS

Phenazine samples were analyzed as previously described (Saunders et al. 2020; McRose and Newman 2021). Briefly, samples were autosampled from 10°C into a Waters LC–MS system (Waters e2695 Separations Module, 2998 PDA Detector, QDA Detector) with 10 μL injections onto a reverse phase C‐18 column (XBridge #186006035) with a running gradient of 98% H2O to 88% acetonitrile over 11 min (run times were 20 min total) with 2% methanol throughout. UV–Vis and positive MS scans were acquired for each run. PYO and 1‐OHPHZ were distinguished by retention time (~3 min and ~7 min, respectively), detected at 364 nm, and manually verified by examining masses 211.2 (PYO) and 197.2 (1‐OHPHZ). ·NO‐reacted phenazines were detected at 364 nm and had predicted masses of 240.2 (PYO‐NO) and 226.2 (1‐OHPHZ 1 & 2). Peaks were automatically mapped in the UV–Vis channels by retention time and the LC trace at 364 nm was exported as a text file from the Empower software.

### 4.13 UV–VIS Spectroscopy

Phenazine stocks were diluted to 100 μM into either 1× PBS for neutral buffering or 20 mM HCl for acidic conditions. Samples were placed into a quartz 96 well plate and absorbance was measured between 200 and 800 nm with 5 nm steps and no shaking on a SpectraMax Multi‐Mode microplate reader.

### 4.14 Reaction Mechanism Calculations

All geometry optimizations of intermediates and transition states were achieved using the spin unrestricted uwb97xD (Chai and Head‐Gordon 2008)/cc‐pvDZ (Hill and Peterson 2017) method, in water using the CPCM solvent model (Klamt and Schüürmann 1993; Persico 1994; Andzelm and Klamt 1995; Barone 1998; Cossi et al. 2003) as implemented in Gaussian16 (Frisch et al. 2016). All calculations used the “guess = mix,always” keywords and “opt = noeigen” was implemented for transition states. Frequency calculations were also conducted at the same level of theory to obtain vibrational frequencies to determine the identity of the stationary points as intermediates (no imaginary frequencies) or as transition states (only one imaginary frequency), as well as obtaining the thermochemistry: enthalpy (DH) and free energy (DG) at the temperature of 298 K. All spin and charge densities were done using the “pop = nbo” (Foster 1980) keyword at the uwb97xD/6–311 + g(d,p)‐cpcm(H_2_O)//uwb97xD//cc‐pvDZ‐cpcm(H_2_O) level of theory. Extensive conformational analysis was performed using CREST (Foster 1980; Pracht et al. 2024; Grimme 2019) version 3.0.2 with XTB (Grimme et al. 2017; Bannwarth et al. 2019) version 6.7.1 and only the lowest‐energy species are shown and discussed. All structural figures were generated with CYLview (Legault 2009). Distances in structural figures are shown in Å and energies are in kcal/mol. Single Point energy corrections were carried out further with the following methods.

- uwb97xD/6–311 + g(d,p)‐CPCM(H_2_O)//uwb97xD/cc‐pvDZ‐cpcm(H_2_O).
- uwb97xD/aug‐cc‐pvTZ‐CPCM(H_2_O)//uwb97xD/cc‐pvDZ‐cpcm(H_2_O).
- uwb97xD/aug‐cc‐pvQZ‐CPCM(H_2_O)//uwb97xD/cc‐pvDZ‐cpcm(H_2_O).

## Author Contributions

**Zachery R. Lonergan:** conceptualization, investigation, funding acquisition, writing – original draft, methodology, visualization, writing – review and editing. **Dianne K. Newman:** conceptualization, funding acquisition, writing – review and editing, project administration, supervision. **Jinyang Li:** methodology, writing – review and editing. **Stuart J. Conway:** conceptualization, methodology, writing – review and editing, funding acquisition, supervision. **Matthew Scurria:** investigation, writing – review and editing, methodology, visualization. **Sarah L. Weisflog:** investigation, writing – review and editing. **Osvaldo Gutierrez:** methodology, writing – review and editing, supervision, funding acquisition. **Korbinian Thalhammer:** methodology, writing – review and editing.

## Funding

This work was supported by Jane Coffin Childs Memorial Fund for Medical Research, National Institutes of Health (NIH) K22AI182146, NIH R01HL152190, NIH 2R01AI27850‐06A1, NIH R35GM137797.

## Ethics Statement

The authors have nothing to report.

## Conflicts of Interest

The authors declare no conflicts of interest.

## Supporting information


**Figure S1:** Nitric oxide is acutely toxic to *P. aeruginosa* , and PYO does not negatively impact *Pa* growth. (A) CFU/mL after 1 h exposure to ⋅NO delivered via the small molecule donor DEA‐NONOate; *n* = 2 biological replicates, mean ± SD. (B) Representative growth monitored by absorbance at 500 nm of wildtype (WT) *Pa* with 100 μM PYO supplemented at the start of growth; *n* = 3 technical replicates from 1 of 3 biological replicates, mean ± SD. (C) Growth monitored by absorbance at 500 nm of Δ*phz Pa* with 100 μM PYO supplemented at the start of growth; *n* = 3 technical replicates from 1 of 3 biological replicates, mean ± SD.
**Figure S2:** Nitric oxide reacts with phenazines to yield chemically distinct redox‐active metabolites. (A) Chromatogram at 364 nm with masses of 500 μM PYO reacted with ⋅NO in oxic, acidic conditions indicated delivered via 3 mM DEA‐NONOate. (B) Chromatogram at 364 nm of LC/MS analysis of 500 μM 1‐OHPHZ reacted with ⋅NO delivered via 3 mM DEA‐NONOate in oxic and anoxic conditions, and at acidic or neutral pH. (C) UV–Vis analysis of 100 μM PYO after ⋅NO reactions performed in acidic or neutral conditions, with ⋅NO gas delivered via continuous flow (see Section 4). (D) UV–Vis analysis of 100 μM 1‐OHPHZ after ⋅NO reactions performed in acidic or neutral conditions ⋅NO gas delivered via continuous flow (see Section 4). (E) Incubation of *Pa* with 100 μM PYO or PYO reacted with ⋅NO (PYO‐NO).
**Figure S3:** Spectral analysis of 1‐OHPHZ following reactivity with NO. FTIR analysis of 1‐OHPHZ and derivatives.
**Figure S4:** PYO‐NO reactivity is acutely toxic to *P. aeruginosa* . (A) Bacterial survival reported as CFU/mL of WT and Δ*fhp Pa* after incubation with 100 μM PYO ±1.5 mM DEA‐NONOate (‘⋅NO’) for 1 h; *n* = 4 biological replicates, mean ± SD. (B) Bacterial survival reported as CFU/mL of *Pa* after incubation with 100 μM PYO ±1.5 mM DEA‐NONOate (‘NO’), with the addition of ⋅NO scavenger carboxy‐PTIO (PTIO) after 1 h of exposure; *n* = 2 biological replicates, mean ± SD. ***p < 0.001, one‐way ANOVA with Tukey multiple comparisons vs. buffer.
**Figure S5:** Decay rate of DEA‐NONOate. Rate of decay was calculated based on commercially‐available metrics and starting concentration of DEA‐NONOate of 1.5 mM, which as a half‐life of 16 min at 22°C–25°C, where *k* = 4.332 × 10^−2^/min. (A) The change in total DEA‐NONOate concentration with respect to time, or d[DEA]/dt = k*[1.5 mM]. (B) Cumulative ⋅NO concentration with respect to time, or d[NO]/dt = 1.5*k[1.5 mM]. The factor 1.5 accounts for the release of 1.5 mol of NO per mole of DEA‐NONOate and assumes complete release. This plot does not account for NO consumption by cells or its reactivity with biomolecules or O_2_, and therefore represents the upper bound of cumulative ⋅NO over time. (C) Rate of ⋅NO delivery, calculated by taking the step‐wise derivative of Panel A.
**Figure S6:** Energetic pathway of 1,2‐addition of Nitric oxide to 1‐Hydroxyphenazine calculated at different computational methods.
**Figure S7:** Energetic pathway of 1,4‐addition of Nitric oxide to 1‐Hydroxyphenazine calculated at different computational methods.
**Figure S8:** Alternative mechanism of 1,2‐addition of Nitric oxide to 1‐HydroxyPhenazine.
**Figure S9:** Alternative mechanism of 1,4‐addition of Nitric oxide to 1‐Hydroxyphenazine.
**Figure S10:** Alternative mechanism of 1,2‐addition of Nitric oxide to 1‐HydroxyPhenazine.
**Figure S11:** Alternative mechanism of 1,4‐addition of Nitric oxide to 1‐HydroxyPhenazine.
**Figure S12:** NBO charge density of 1‐Hydroxyphenazine at the uwb97xD/6–311 + g (d,p)‐cpcm(H_2_O)//uwb97xD//cc‐pvDZ‐cpcm(H_2_O) level of theory.
**Data S1:** Supplementary computational details.
**Table S1:** Rf values for phenazine reactivity.
**Table S2:** Cartesian coordinates (xyz format) and energies of all the structures involved in each reaction mechanism studied calculated at the uwb97xD//cc‐pvDZ‐cpcm (H_2_O) level of theory.

## Data Availability

The data supporting the findings of this study are available from the corresponding authors upon reasonable request.
