# Supplementary material for: Nitric Oxide Tunes Secreted Metabolite Bioactivity
Source: Mol Microbiol. 2026 Jun 8;126(2):145–60. doi: 10.1111/mmi.70083 (PMC13251328; doi:10.1111/mmi.70083)
Supplement: Supplementary file 1 — Figure S1: Nitric oxide is acutely toxic to P. aeruginosa , and PYO does not negatively impact Pa growth. (A) CFU/mL after 1 h exposure to ⋅NO delivered via the small molecule donor DEA‐NONOate; n = 2 biological replicates, mean ± SD. (B) Representative growth monitored by absorbance at 500 nm of wildtype (WT) Pa with 100 μM PYO supplemented at the start of growth; n = 3 technical replicates from 1 of 3 biological replicates, mean ± SD. (C) Growth monitored by absorbance at 500 nm of Δphz Pa with 100 μM PYO supplemented at the start of growth; n = 3 technical replicates from 1 of 3 biological replicates, mean ± SD. Figure S2: Nitric oxide reacts with phenazines to yield chemically distinct redox‐active metabolites. (A) Chromatogram at 364 nm with masses of 500 μM PYO reacted with ⋅NO in oxic, acidic conditions indicated delivered via 3 mM DEA‐NONOate. (B) Chromatogram at 364 nm of LC/MS analysis of 500 μM 1‐OHPHZ reacted with ⋅NO delivered via 3 mM DEA‐NONOate in oxic and anoxic conditions, and at acidic or neutral pH. (C) UV–Vis analysis of 100 μM PYO after ⋅NO reactions performed in acidic or neutral conditions, with ⋅NO gas delivered via continuous flow (see Section 4). (D) UV–Vis analysis of 100 μM 1‐OHPHZ after ⋅NO reactions performed in acidic or neutral conditions ⋅NO gas delivered via continuous flow (see Section 4). (E) Incubation of Pa with 100 μM PYO or PYO reacted with ⋅NO (PYO‐NO). Figure S3: Spectral analysis of 1‐OHPHZ following reactivity with NO. FTIR analysis of 1‐OHPHZ and derivatives. Figure S4: PYO‐NO reactivity is acutely toxic to P. aeruginosa . (A) Bacterial survival reported as CFU/mL of WT and Δfhp Pa after incubation with 100 μM PYO ±1.5 mM DEA‐NONOate (‘⋅NO’) for 1 h; n = 4 biological replicates, mean ± SD. (B) Bacterial survival reported as CFU/mL of Pa after incubation with 100 μM PYO ±1.5 mM DEA‐NONOate (‘NO’), with the addition of ⋅NO scavenger carboxy‐PTIO (PTIO) after 1 h of exposure; n = 2 biological replicates, mean ± SD. ***p < 0 [file MMI-126-145-s001.docx]

**Supporting Information**

**Nitric oxide tunes secreted metabolite bioactivity**

Zachery R. Lonergan^a,d^*, Sarah L. Weisflog^a^, Matthew Scurria^b^, Jinyang Li^a^, Korbinian Thalhammer^c^, Osvaldo Gutierrez^b^, Stuart J. Conway^b^*, Dianne K. Newman^a,c^*

^a^ Division of Biology and Biological Engineering, California Institute of Technology, Pasadena, CA 91125

^b^ Department of Chemistry and Biochemistry, University of California, Los Angeles, CA 90095

^c^ Division of Geological and Planetary Sciences, California Institute of Technology, Pasadena, CA 91125

^d^ Present address: Department of Biochemistry and Microbiology, Rutgers University, New Brunswick, NJ 08901

*Corresponding authors: [z.lonergan@rutgers.edu](mailto:z.lonergan@rutgers.edu), [stuartconway@ucla.edu](mailto:stuartconway@ucla.edu), [dkn@caltech.edu](mailto:dkn@caltech.edu)


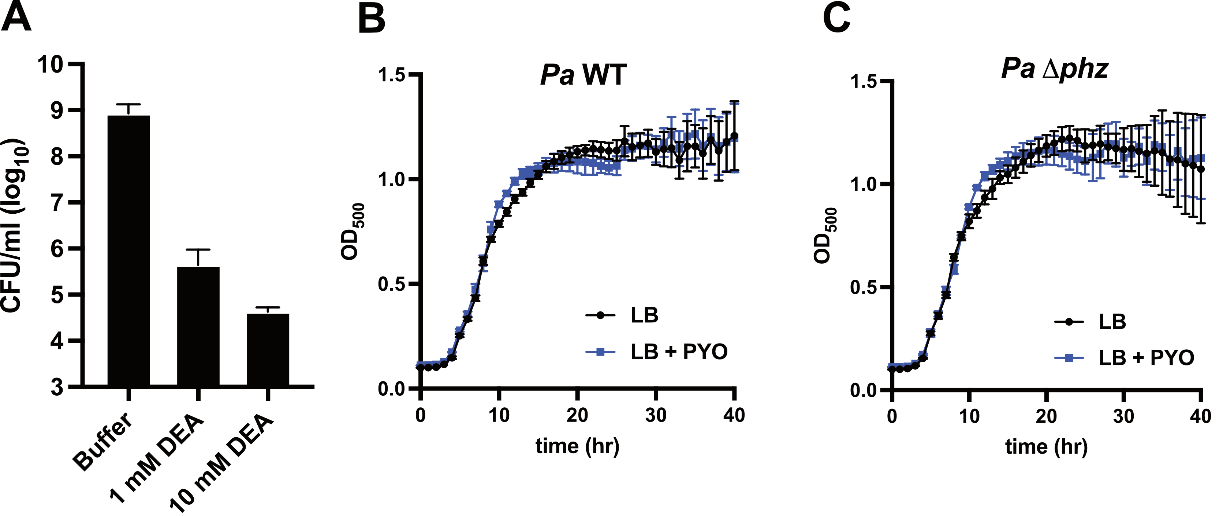


**Figure S1. Nitric oxide is acutely toxic to *P. aeruginosa*, and** **PYO does not negatively impact *Pa* growth.** **A)** CFU/mL after 1 h exposure to ⋅NO delivered via the small molecule donor DEA-NONOate; n = 2 biological replicates, mean +/- SD. **B)** Representative growth monitored by absorbance at 500 nm of wildtype (WT) *Pa* with 100 μM PYO supplemented at the start of growth; n = 3 technical replicates from 1 of 3 biological replicates, mean +/- SD. **C)** Growth monitored by absorbance at 500 nm of Δ*phz* *Pa* with 100 μM PYO supplemented at the start of growth; n = 3 technical replicates from 1 of 3 biological replicates, mean +/- SD.


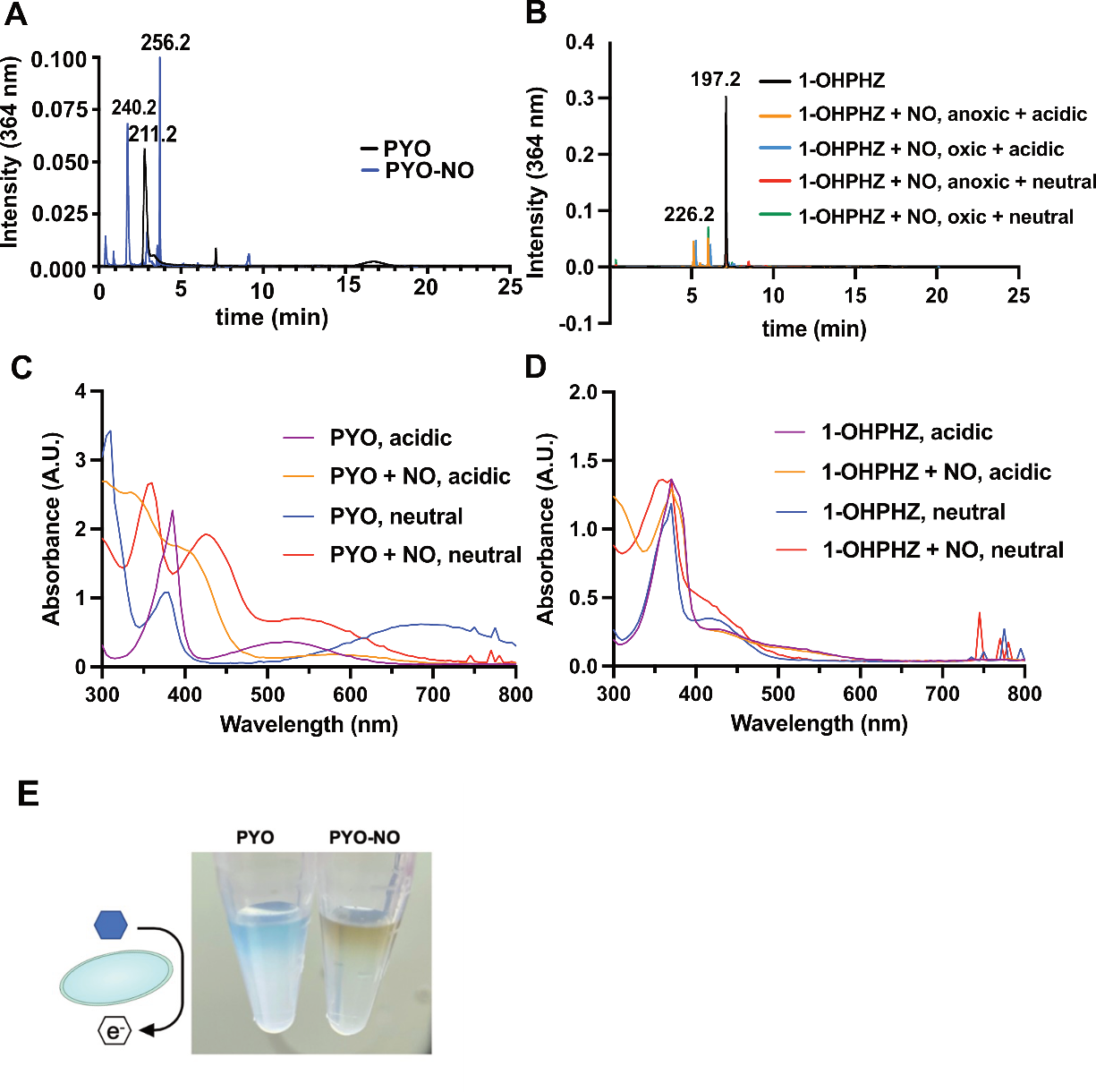


**Figure S2. Nitric oxide reacts with phenazines to yield chemically distinct redox-active metabolites. A)** Chromatogram at 364 nm with masses of 500 μM PYO reacted with ⋅NO in oxic, acidic conditions indicated delivered via 3 mM DEA-NONOate. **B)** Chromatogram at 364 nm of LC/MS analysis of 500 μM 1-OHPHZ reacted with ⋅NO delivered via 3mM DEA-NONOate in oxic and anoxic conditions, and at acidic or neutral pH. **C)** UV-Vis analysis of 100 μM PYO after ⋅NO reactions performed in acidic or neutral conditions, with ⋅NO gas delivered via continuous flow (see Materials and Methods). **D)** UV-Vis analysis of 100 μM 1-OHPHZ after ⋅NO reactions performed in acidic or neutral conditions ⋅NO gas delivered via continuous flow (see Materials and Methods). **E)** Incubation of *Pa* with 100 μM PYO or PYO reacted with ⋅NO (PYO-NO).


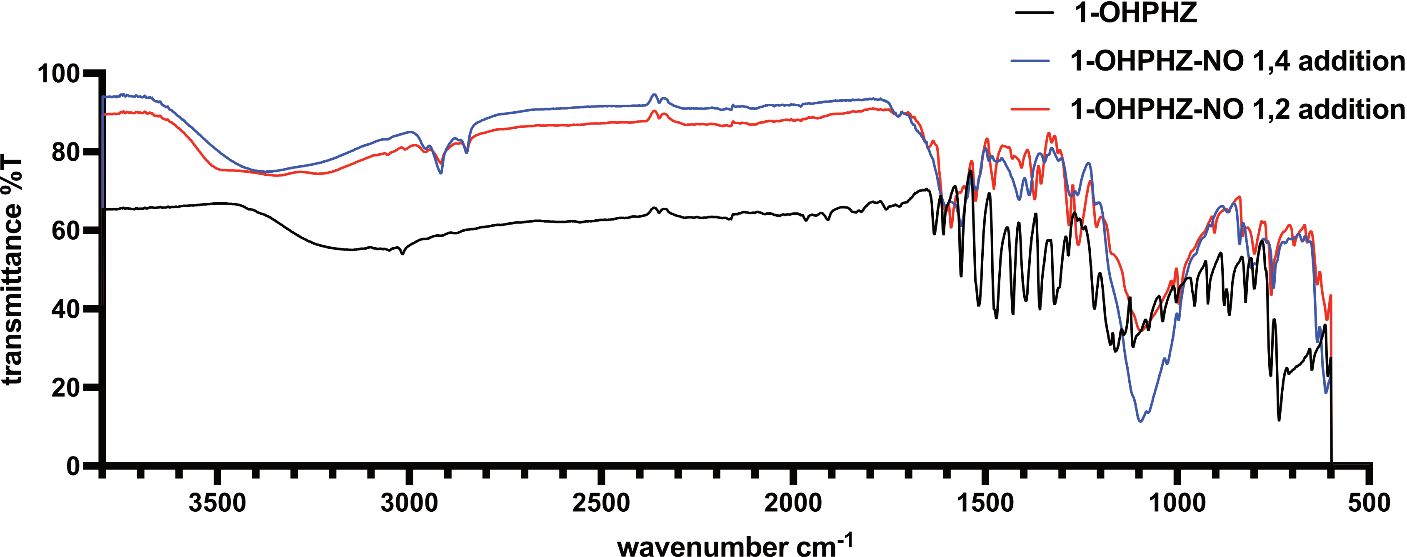


**Figure S3. Spectral analysis of 1-OHPHZ following reactivity with NO.** FTIR analysis of 1-OHPHZ and derivatives.


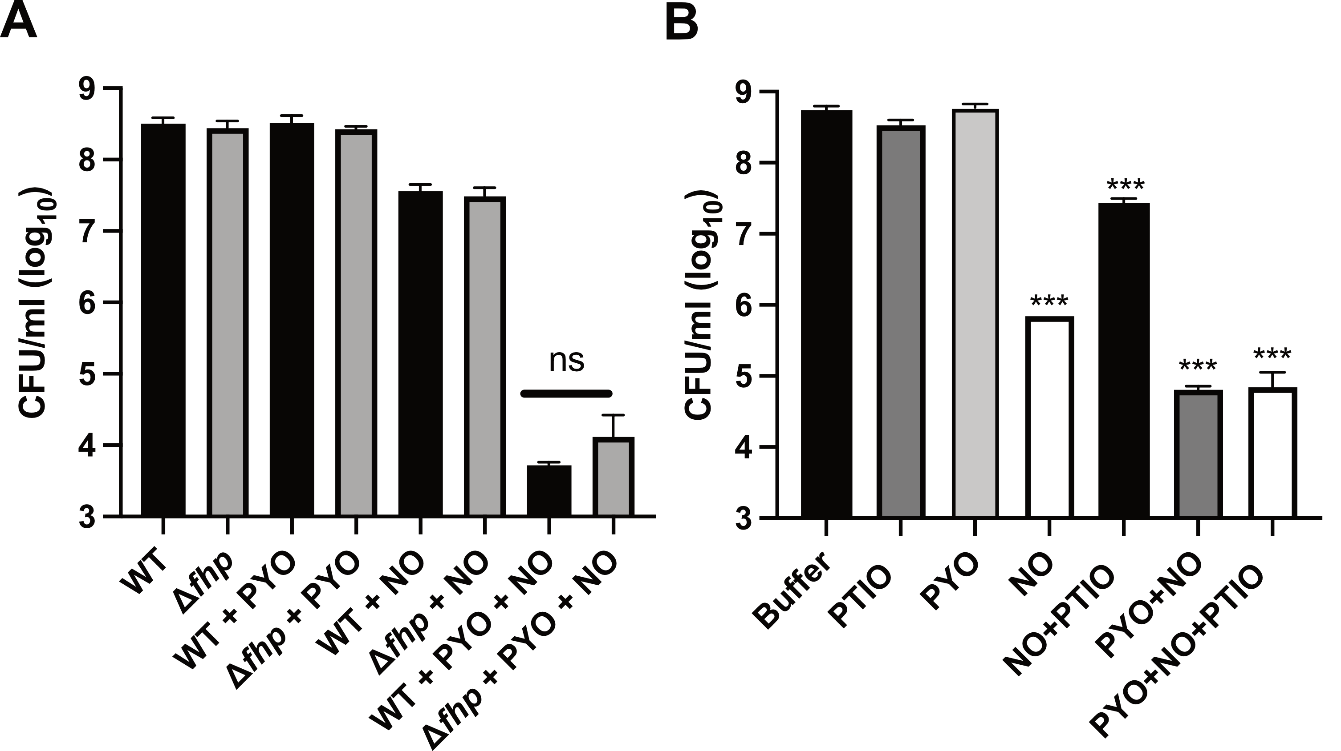


**Figure S4. PYO-NO reactivity is acutely toxic to *P. aeruginosa*. A)** Bacterial survival reported as CFU/mL of WT and Δ*fhp* *Pa* after incubation with 100 μM PYO +/- 1.5 mM DEA-NONOate (‘⋅NO’) for 1 h; n = 4 biological replicates, mean +/- SD. **B)** Bacterial survival reported as CFU/mL of *Pa* after incubation with 100 μM PYO +/- 1.5 mM DEA-NONOate (‘⋅NO’), with the addition of ⋅NO scavenger carboxy-PTIO (PTIO) after 1 h of exposure; n = 2 biological replicates, mean +/- SD. ***p < 0.001, one-way ANOVA with Tukey multiple comparisons vs buffer.

**
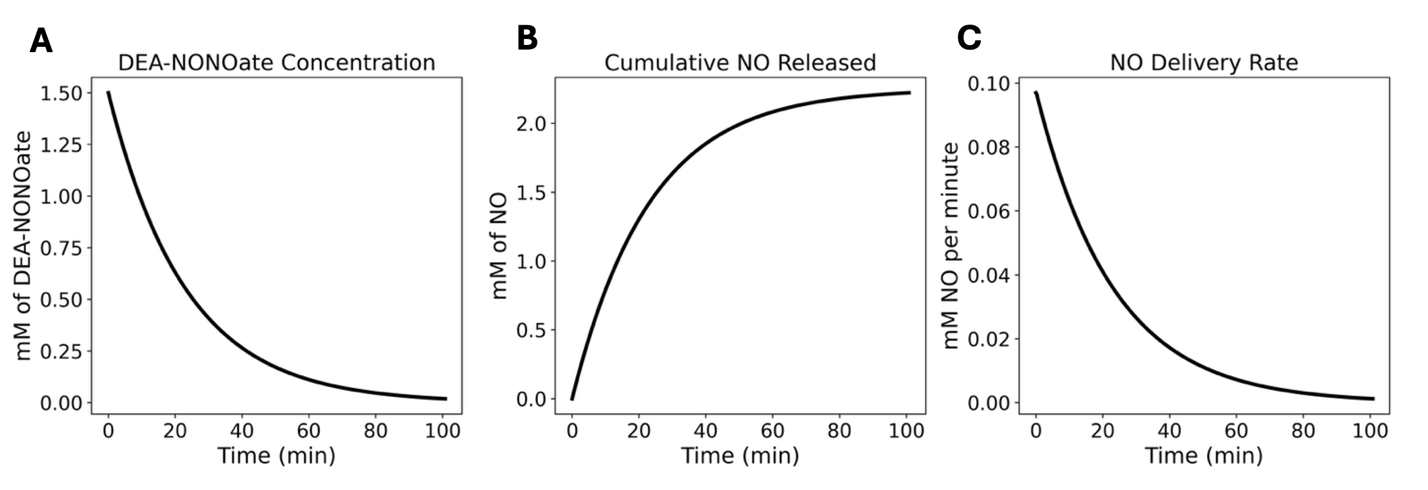
 Figure S5. Decay rate of DEA-NONOate.** Rate of decay was calculated based on commercially available metrics and starting concentration of DEA-NONOate of 1.5 mM, which as a half-life of 16 minutes at 22-25 °C, where *k* = 4.332x10^-2^ min^-1^. **A)** The change in total DEA-NONOate concentration with respect to time, or d[DEA]/dt = *k**[1.5 mM]. **B)** Cumulative ⋅NO concentration with respect to time, or d[NO]/dt = 1.5**k*[1.5 mM]. The factor 1.5 accounts for the release of 1.5 mole of NO per mole of DEA-NONOate and assumes complete release. This plot does not account for NO consumption by cells or its reactivity with biomolecules or O_2_, and therefore represents the upper bound of cumulative ⋅NO over time. **C)** Rate of ⋅NO delivery, calculated by taking the step-wise derivative of Panel A.

**Table S1. Rf values for phenazine reactivity.**

| **Compound** | **Rf** | **Mobile Phase** |
| --- | --- | --- |
| Pyocyanin | 0.79 | 1 |
| Pyocyanin-NO (acidic) | 0.44 | 1 |
| Pyocyanin-NO (neutral) | 0.45 | 1 |
| 1-OHPHZ | 0.86 | 2 |
| 1-OHPHZNO 1,2 (acidic) | 0.55 | 2 |
| 1-OHPHZNO 1,4 (acidic) | 0.36 | 2 |
| 1-OHPHZNO 1,2 (neutral) | 0.55 | 2 |
| 1-OHPHZNO 1,4 (neutral) | 0.35 | 2 |

Mobile Phase 1: 50:50 chloroform:methanol; Mobile Phase 2: 95:5 chloroform:methanol.

**Data S1**

**Supplementary** **Computational Details**

All geometry optimizations of intermediates and transition states were achieved using the spin unrestricted uwb97xD ^Ref1^/cc-pvDZ ^Ref2^ method, in water using the CPCM solvent model ^Ref3^ as implemented in Gaussian16 ^Ref4^. All calculations used the “guess=mix,always” keywords and “opt=noeigen” was implemented for transition states. Frequency calculations were also conducted at the same level of theory to obtain vibrational frequencies to determine the identity of the stationary points as intermediates (no imaginary frequencies) or as transition states (only one imaginary frequency), as well as obtaining the thermochemistry: enthalpy (DH) and free energy (DG) at the temperature of 298 K. All spin and charge densities were done using the “pop=nbo” ^Ref5^ keyword at the uwb97xD/6-311+g(d,p)-cpcm(H_2_O)//uwb97xD//cc-pvDZ-cpcm(H_2_O) level of theory. Extensive conformational analysis was performed using CREST ^Ref6^ version 3.0.2 with XTB ^Ref7^ version 6.7.1 and only the lowest-energy species are shown and discussed. All structural figures were generated with CYLview ^Ref8^. Distances in structural figures are shown in Å and energies are in kcal/mol. Single Point energy corrections were carried out further with the following methods.

1. uwb97xD/6-311+g(d,p)-CPCM(H_2_O)//uwb97xD/cc-pvDZ-cpcm(H_2_O)
2. uwb97xD/aug-cc-pvTZ-CPCM(H_2_O)// uwb97xD/cc-pvDZ-cpcm(H_2_O)
3. uwb97xD/aug-cc-pvQZ-CPCM(H_2_O)// uwb97xD/cc-pvDZ-cpcm(H_2_O)

1. (a) J.-D. Chai and M. Head-Gordon, “Systematic optimization of long-range corrected hybrid density functionals,” *J. Chem. Phys.*, **128** (2008) 084106. (b) J.-D. Chai and M. Head-Gordon, “Long-range corrected hybrid density functionals with damped atom-atom dispersion corrections,” *Phys. Chem. Chem. Phys.*, **10** (2008) 6615-20

2. T. H. Dunning Jr., “Gaussian basis sets for use in correlated molecular calculations. I. The atoms boron through neon and hydrogen,” *J. Chem. Phys.*, **90** (1989) 1007-23

3. (a) Klamt, A.; Schüürmann, G. COSMO: a new approach to dielectric screening in solvents with explicit expressions for the screening energy and its gradient. *J. Chem. Soc. Perkin Trans. 2* **1993**, *0*, 799-805. (b) Tomasi, J.; Persico, M. Molecular Interactions in Solution: An Overview of Methods Based on Continuous Distributions of the Solvent. *Chem. Rev.* **1994**, *94*, 2027-2094. (c) Andzelm, J.; Kölmel, C.; Klamt, A. Incorporation of solvent effects into density functional calculations of molecular energies and geometries*. J. Chem. Phys.* **1995**, *103*, 9312-9320. (d) Barone, V.; Cossi, M. Quantum Calculation of Molecular Energies and Energy Gradients in Solution by a Conductor Solvent Model. *J. Phys. Chem. A* **1998**, *102*, 1995-2001. (e) Cossi, M.; Rega, N.; Scalmani, G.; Barone, V. Energies, structures, and electronic properties of molecules in solution with the C-PCM solvation model. *J. Comput. Chem.* **2003**, *24*, 669-681.

4. Gaussian 16, Revision C.01, Frisch, M. J.; Trucks, G. W.; Schlegel, H. B.; Scuseria, G. E.; Robb, M. A.; Cheeseman, J. R.; Scalmani, G.; Barone, V.; Petersson, G. A.; Nakatsuji, H.; Li, X.; Caricato, M.; Marenich, A. V.; Bloino, J.; Janesko, B. G.; Gomperts, R.; Mennucci, B.; Hratchian, H. P.; Ortiz, J. V.; Izmaylov, A. F.; Sonnenberg, J. L.; Williams-Young, D.; Ding, F.; Lipparini, F.; Egidi, F.; Goings, J.; Peng, B.; Petrone, A.; Henderson, T.; Ranasinghe, D.; Zakrzewski, V. G.; Gao, J.; Rega, N.; Zheng, G.; Liang, W.; Hada, M.; Ehara, M.; Toyota, K.; Fukuda, R.; Hasegawa, J.; Ishida, M.; Nakajima, T.; Honda, Y.; Kitao, O.; Nakai, H.; Vreven, T.; Throssell, K.; Montgomery, J. A., Jr.; Peralta, J. E.; Ogliaro, F.; Bearpark, M. J.; Heyd, J. J.; Brothers, E. N.; Kudin, K. N.; Staroverov, V. N.; Keith, T. A.; Kobayashi, R.; Normand, J.; Raghavachari, K.; Rendell, A. P.; Burant, J. C.; Iyengar, S. S.; Tomasi, J.; Cossi, M.; Millam, J. M.; Klene, M.; Adamo, C.; Cammi, R.; Ochterski, J. W.; Martin, R. L.; Morokuma, K.; Farkas, O.; Foresman, J. B.; Fox, D. J. Gaussian, Inc., Wallingford CT, 2016.

5. J. P. Foster and F. Weinhold, “Natural hybrid orbitals,” *J. Am. Chem. Soc.*, 102 (1980) 7211-18

6. (a) Pracht, P.; Bohle, F.; Grimme, S.; *Automated exploration of the low-energy chemical space with fast quantum chemical methods*, *Phys. Chem. Chem. Phys.*, **2020**, *22*, 7169-7192. (b) Pracht, P.; Grimme, S.; Bannwarth, C.; Bohle, F.; Ehlert, S.; Feldmann, G.; Gorges, J.; Müller, M.; Neudecker, T.; Plett, C.; Spicher, S.; Steinbach, P.; Wesołowski, P.A.; Zeller, F.; *CREST — A program for the exploration of low-energy molecular chemical space*, *J. Chem. Phys.*, **2024**, *160*, 114110. (c) Grimme, S.; *Exploration of Chemical Compound, Conformer, and Reaction Space with Meta-Dynamics Simulations Based on Tight-Binding Quantum Chemical Calculations*, *J. Chem. Theory Comput.*, **2019**, *15 (5)*, 2847-2862.

7. (a) Grimme, S.; Bannwarth, C.; Shushkov, P.; *A Robust and Accurate Tight-Binding Quantum Chemical Method for Structures, Vibrational Frequencies, and Noncovalent Interactions of Large Molecular Systems Parameterized for All spd-Block Elements (Z = 1-86).* *J. Chem. Theory Comput.*, **2017**, *13 (5)*, 1989-2009. (b) Bannwarth, C.; Ehlert, S.; Grimme, S.; *GFN2-xTB — An Accurate and Broadly Parametrized Self-Consistent Tight-Binding Quantum Chemical Method with Multipole Electrostatics and Density-Dependent Dispersion Contributions* *J. Chem. Theory Comput.* **2019**, *15 (3)*, 1652–1671.

8. Legault, C. Y. (2009) CYLview, 1.0b, Université de Sherbrooke: Sherbrooke, Canada, <http://www.cylview.org>.

**Figure S6**. Energetic pathway of 1,2-addition of Nitric oxide to 1-Hydroxyphenazine calculated at different computational methods.

- *^2^TS_3-3’_ represents the tautomerization between intermediates ^2^3 and ^2^3’*

**Figure S7**. Energetic pathway of 1,4-addition of Nitric oxide to 1-Hydroxyphenazine calculated at different computational methods.

- *^4^TS_3-3’_ represents the tautomerization between intermediates ^4^3 and ^4^3’*

**Figure S8**. Alternative mechanism of 1,2-addition of Nitric oxide to 1-HydroxyPhenazine.

- *Addition of Nitric Oxide to the Phenazine ring was determined to be a completely uphill process.*

**Figure S9**. Alternative mechanism of 1,4-addition of Nitric oxide to 1-Hydroxyphenazine.

- *Addition of Nitric Oxide to the Phenazine ring was determined to be a completely uphill process.*

**

**Figure S10**. Alternative mechanism of 1,2-addition of Nitric oxide to 1-HydroxyPhenazine.

- *^2^TS_2-2’_ represents the tautomerization between intermediates ^2^2 and ^2^2’*
- *^2^TS_2’-3*_ represents the tautomerization between intermediates ^2^2’ and ^2^3**

**Figure S11**. Alternative mechanism of 1,4-addition of Nitric oxide to 1-HydroxyPhenazine.

- *^4^TS_2-2’_ represents the tautomerization between intermediates ^4^2 and ^4^2’*
- *^4^TS_2’-3*_ represents the tautomerization between intermediates ^4^2’ and ^4^3**

**Figure S12**. NBO charge density of 1-Hydroxyphenazine at the uwb97xD/6-311+g(d,p)-cpcm(H_2_O)//uwb97xD//cc-pvDZ-cpcm(H_2_O) level of theory.

- *The 2 and 4 positions represent the most nucleophilic sites of the ring.*

**Table S2.** Cartesian coordinates (xyz format) and energies of all the structures involved in each reaction mechanism studied calculated at the uwb97xD//cc-pvDZ-cpcm(H_2_O) level of theory.

**1**

E(scf) = -906.381162860 a.u.

| C | -4.095080 | -0.375167 | -0.467441 |
| --- | --- | --- | --- |
| C | -2.995123 | -0.788045 | -1.162784 |
| C | -2.778965 | 1.176761 | 0.862851 |
| C | -3.986394 | 0.617811 | 0.556780 |
| H | -5.073578 | -0.803589 | -0.689306 |
| H | -3.056063 | -1.543967 | -1.946593 |
| H | -2.671872 | 1.934015 | 1.640412 |
| H | -4.883582 | 0.926231 | 1.095273 |
| C | -1.709433 | -0.229056 | -0.874135 |
| C | -1.604951 | 0.769720 | 0.156443 |
| C | 0.541206 | -0.102392 | -1.261422 |
| C | 1.729644 | -0.496780 | -1.947797 |
| C | 2.925043 | 0.077472 | -1.605373 |
| C | 3.035191 | 1.054220 | -0.574035 |
| C | 1.916123 | 1.457707 | 0.108544 |
| C | 0.636314 | 0.887111 | -0.230215 |
| H | 1.651383 | -1.250665 | -2.730760 |
| H | 3.834818 | -0.222824 | -2.127660 |
| H | 4.004471 | 1.483868 | -0.320833 |
| N | -0.420761 | 1.314119 | 0.462348 |
| N | -0.642576 | -0.649873 | -1.567060 |
| O | 1.956516 | 2.373396 | 1.086983 |
| H | 1.036414 | 2.472539 | 1.396156 |
| N | 2.231258 | -1.320397 | 1.506419 |
| N | 0.404999 | -1.216261 | 2.046500 |
| O | -0.080574 | -2.012772 | 1.368440 |
| O | 2.182325 | -2.133738 | 0.691748 |


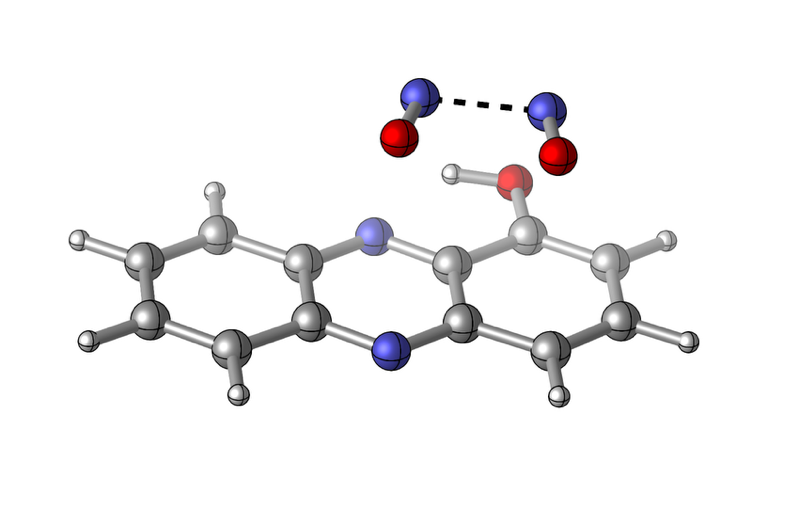


Zero-point correction= 0.190614 (Hartree/Particle)

Thermal correction to Energy= 0.206516

Thermal correction to Enthalpy= 0.207460

Thermal correction to Gibbs Free Energy= 0.145511

Sum of electronic and zero-point Energies= -906.190549

Sum of electronic and thermal Energies= -906.174647

Sum of electronic and thermal Enthalpies= -906.173703

Sum of electronic and thermal Free Energies= -906.235652

**^2^TS_2-3_**

E(scf) = -906.346379561 a.u.

ν_min_ = -546.0 cm^-1^

| C | -0.585584 | -0.831972 | 0.010453 |
| --- | --- | --- | --- |
| C | -1.548264 | 0.140499 | 0.114139 |
| C | -2.191944 | -2.425595 | -0.868689 |
| C | -0.909938 | -2.124509 | -0.484629 |
| H | 0.441135 | -0.616375 | 0.309375 |
| H | -1.320996 | 1.138498 | 0.490773 |
| H | -2.463428 | -3.410237 | -1.251180 |
| H | -0.127389 | -2.880950 | -0.557930 |
| C | -2.886434 | -0.137806 | -0.273032 |
| C | -3.209639 | -1.439017 | -0.770501 |
| C | -5.052780 | 0.533288 | -0.527951 |
| C | -6.117352 | 1.521425 | -0.449117 |
| C | -7.383515 | 1.208419 | -0.783992 |
| C | -7.778550 | -0.145242 | -1.211217 |
| C | -6.721352 | -1.074683 | -1.424717 |
| C | -5.367858 | -0.775053 | -1.018337 |
| H | -5.845067 | 2.523513 | -0.116341 |
| H | -8.173115 | 1.957316 | -0.722453 |
| N | -4.475079 | -1.738633 | -1.146751 |
| N | -3.823189 | 0.840173 | -0.163116 |
| O | -6.993657 | -2.264637 | -1.926634 |
| H | -6.169395 | -2.789518 | -1.855668 |
| H | -8.642025 | -0.212330 | -1.876600 |
| N | -8.466087 | -1.025109 | 0.131351 |
| O | -9.061411 | -2.043736 | -0.142816 |
| N | -7.241712 | -1.238322 | 1.107847 |
| O | -7.313937 | -2.315499 | 1.582163 |

Zero-point correction= 0.190319 (Hartree/Particle)

Thermal correction to Energy= 0.204846

Thermal correction to Enthalpy= 0.205790

Thermal correction to Gibbs Free Energy= 0.148215

Sum of electronic and zero-point Energies= -906.156060

Sum of electronic and thermal Energies= -906.141534

Sum of electronic and thermal Enthalpies= -906.140589

Sum of electronic and thermal Free Energies= -906.198165

**^2^2**

E(scf) = -906.358671158 a.u.

| C | 4.848731 | 0.252082 | 0.553838 |
| --- | --- | --- | --- |
| C | 3.823889 | 1.162580 | 0.395969 |
| C | 3.339720 | -1.591419 | 0.103220 |
| C | 4.602748 | -1.132751 | 0.405761 |
| H | 5.854778 | 0.599174 | 0.793519 |
| H | 3.990004 | 2.235496 | 0.504010 |
| H | 3.129346 | -2.655447 | -0.014654 |
| H | 5.422215 | -1.841824 | 0.533089 |
| C | 2.519911 | 0.720619 | 0.086797 |
| C | 2.269776 | -0.676293 | -0.061647 |
| C | 0.329484 | 1.198098 | -0.349641 |
| C | -0.785377 | 2.127075 | -0.542276 |
| C | -2.028331 | 1.702244 | -0.800509 |
| C | -2.391869 | 0.248746 | -0.932180 |
| C | -1.222360 | -0.666470 | -0.813330 |
| C | 0.071187 | -0.216236 | -0.490767 |
| H | -0.558185 | 3.191209 | -0.465362 |
| H | -2.845948 | 2.412168 | -0.933034 |
| N | 1.026801 | -1.141156 | -0.355925 |
| N | 1.520324 | 1.653649 | -0.069717 |
| O | -1.453107 | -1.964803 | -0.988080 |
| H | -0.597171 | -2.409769 | -0.820114 |
| H | -2.936724 | 0.065509 | -1.870898 |
| N | -3.386684 | -0.138996 | 0.116407 |
| O | -4.376697 | -0.824081 | -0.137355 |
| N | -2.995312 | 0.177683 | 1.445154 |
| O | -3.780105 | -0.239171 | 2.249260 |


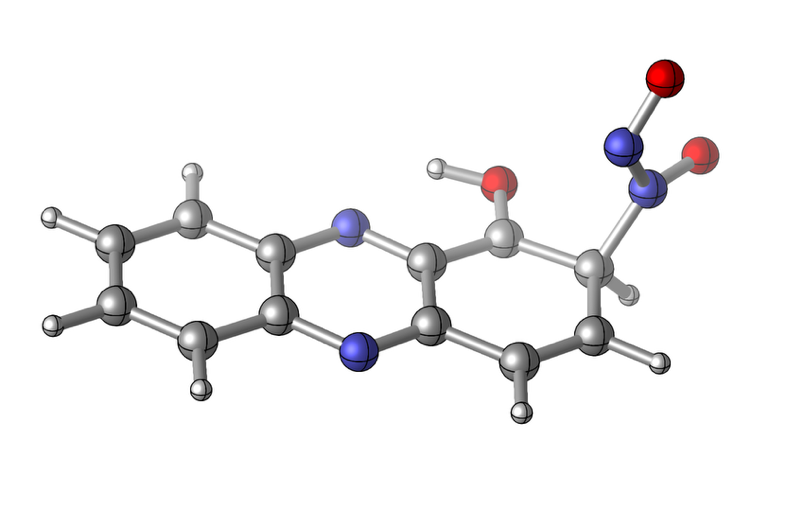


Zero-point correction= 0.192243 (Hartree/Particle)

Thermal correction to Energy= 0.206799

Thermal correction to Enthalpy= 0.207743

Thermal correction to Gibbs Free Energy= 0.149144

Sum of electronic and zero-point Energies= -906.166428

Sum of electronic and thermal Energies= -906.151872

Sum of electronic and thermal Enthalpies= -906.150928

Sum of electronic and thermal Free Energies= -906.209527

**^2^TS_2-3_**

E(scf) = -906.343441288 a.u.

ν_min_ = -259.0 cm^-1^

| C | 0.080045 | 1.052552 | 0.002202 |
| --- | --- | --- | --- |
| C | -0.900422 | 2.015746 | 0.108970 |
| C | -1.507056 | -0.523608 | -0.934610 |
| C | -0.227545 | -0.224965 | -0.523403 |
| H | 1.099196 | 1.272990 | 0.322944 |
| H | -0.687167 | 3.007890 | 0.509796 |
| H | -1.764300 | -1.502411 | -1.342249 |
| H | 0.558371 | -0.977763 | -0.602465 |
| C | -2.222789 | 1.736615 | -0.303725 |
| C | -2.533558 | 0.449150 | -0.833184 |
| C | -4.387524 | 2.411752 | -0.577779 |
| C | -5.458478 | 3.405308 | -0.508474 |
| C | -6.717455 | 3.109212 | -0.863871 |
| C | -7.142742 | 1.743029 | -1.290156 |
| C | -6.026591 | 0.822488 | -1.529219 |
| C | -4.705211 | 1.105433 | -1.103139 |
| H | -5.182513 | 4.403828 | -0.166124 |
| H | -7.499693 | 3.867271 | -0.802641 |
| N | -3.794879 | 0.141916 | -1.237349 |
| N | -3.176645 | 2.718155 | -0.189335 |
| O | -6.304629 | -0.374992 | -2.041502 |
| H | -5.470778 | -0.884885 | -1.984520 |
| H | -7.880628 | 1.748927 | -2.104759 |
| N | -7.905279 | 1.065552 | -0.087844 |
| O | -8.553843 | 0.098483 | -0.390388 |
| N | -6.544196 | 0.348410 | 1.287748 |
| O | -6.951125 | -0.707290 | 1.431226 |

Zero-point correction= 0.189535 (Hartree/Particle)

Thermal correction to Energy= 0.204358

Thermal correction to Enthalpy= 0.205302

Thermal correction to Gibbs Free Energy= 0.146643

Sum of electronic and zero-point Energies= -906.153906

Sum of electronic and thermal Energies= -906.139084

Sum of electronic and thermal Enthalpies= -906.138139

Sum of electronic and thermal Free Energies= -906.196798

**^2^3**

E(scf) = -776.483897779 a.u.

| C | 4.396090 | 0.266549 | 0.335634 |
| --- | --- | --- | --- |
| C | 3.358863 | 1.174143 | 0.288629 |
| C | 2.861229 | -1.574615 | -0.029403 |
| C | 4.143425 | -1.116390 | 0.174649 |
| H | 5.417903 | 0.613313 | 0.496290 |
| H | 3.529659 | 2.245210 | 0.407595 |
| H | 2.645281 | -2.636685 | -0.154992 |
| H | 4.973686 | -1.823350 | 0.213021 |
| C | 2.033190 | 0.732569 | 0.082077 |
| C | 1.777065 | -0.661942 | -0.078308 |
| C | -0.189204 | 1.206366 | -0.152037 |
| C | -1.317115 | 2.132225 | -0.256796 |
| C | -2.575503 | 1.692429 | -0.407846 |
| C | -2.931280 | 0.243789 | -0.388666 |
| C | -1.775791 | -0.653908 | -0.510334 |
| C | -0.452761 | -0.207277 | -0.292838 |
| H | -1.083557 | 3.197570 | -0.227471 |
| H | -3.400836 | 2.401021 | -0.490592 |
| N | 0.514822 | -1.126636 | -0.273237 |
| N | 1.022632 | 1.662606 | 0.030020 |
| O | -2.010447 | -1.952419 | -0.692173 |
| H | -1.139577 | -2.391622 | -0.609206 |
| H | -3.755206 | -0.026992 | -1.062959 |
| N | -3.466763 | -0.106646 | 1.074154 |
| O | -4.195984 | -1.046002 | 1.070072 |


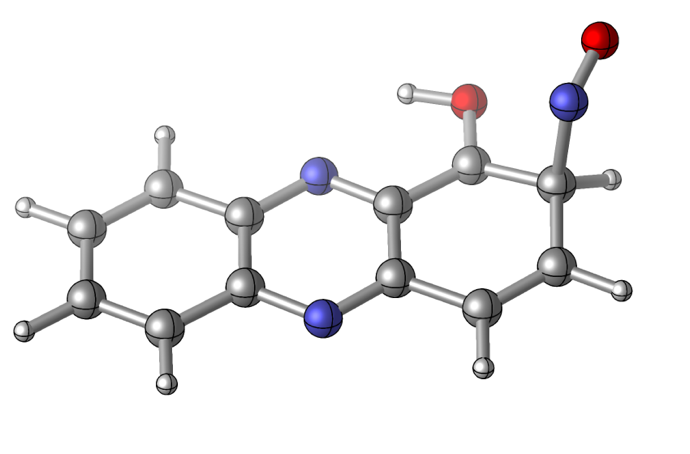


Zero-point correction= 0.182890 (Hartree/Particle)

Thermal correction to Energy= 0.195597

Thermal correction to Enthalpy= 0.196541

Thermal correction to Gibbs Free Energy= 0.142617

Sum of electronic and zero-point Energies= -776.301008

Sum of electronic and thermal Energies= -776.288301

Sum of electronic and thermal Enthalpies= -776.287357

Sum of electronic and thermal Free Energies= -776.341281

**^2^TS_3-3’_**

E(scf) = -852.893830909 a.u.

ν_min_ = -1011.4 cm^-1^

| C | 0.042159 | 0.085430 | -0.002905 |
| --- | --- | --- | --- |
| C | -0.859420 | 1.116641 | 0.101852 |
| C | -1.718331 | -1.567742 | 0.212055 |
| C | -0.388727 | -1.266889 | 0.052382 |
| H | 1.103799 | 0.302769 | -0.129764 |
| H | -0.547215 | 2.160929 | 0.062478 |
| H | -2.076519 | -2.596954 | 0.258188 |
| H | 0.347044 | -2.067657 | -0.032707 |
| C | -2.240436 | 0.835775 | 0.265470 |
| C | -2.672570 | -0.521194 | 0.321439 |
| C | -4.394661 | 1.549518 | 0.510776 |
| C | -5.380655 | 2.620830 | 0.646893 |
| C | -6.691413 | 2.352162 | 0.702329 |
| C | -7.225060 | 0.973683 | 0.540759 |
| C | -6.258431 | -0.119824 | 0.755090 |
| C | -4.822454 | 0.178857 | 0.558667 |
| H | -4.993393 | 3.638977 | 0.702695 |
| H | -7.421925 | 3.157129 | 0.792887 |
| N | -3.983993 | -0.831121 | 0.479000 |
| N | -3.122986 | 1.865364 | 0.371878 |
| O | -6.606377 | -1.307467 | 0.982282 |
| N | -7.456225 | 0.744619 | -0.997577 |
| O | -8.334116 | -0.151131 | -1.221364 |
| H | -7.840519 | -1.512477 | 0.967076 |
| O | -8.955672 | -1.632599 | 0.664917 |

Zero-point correction= 0.203767 (Hartree/Particle)

Thermal correction to Energy= 0.217685

Thermal correction to Enthalpy= 0.218629

Thermal correction to Gibbs Free Energy= 0.162354

Sum of electronic and zero-point Energies= -852.690064

Sum of electronic and thermal Energies= -852.676146

Sum of electronic and thermal Enthalpies= -852.675202

Sum of electronic and thermal Free Energies= -852.731477

**^2^3’**

E(scf) = -776.493289570 a.u.

| C | -4.498926 | 0.203047 | -0.174986 |
| --- | --- | --- | --- |
| C | -3.487497 | 1.131568 | -0.159786 |
| C | -2.921929 | -1.628788 | 0.017638 |
| C | -4.216363 | -1.187065 | -0.086020 |
| H | -5.536094 | 0.531667 | -0.256388 |
| H | -3.686031 | 2.201773 | -0.227085 |
| H | -2.675817 | -2.688939 | 0.087658 |
| H | -5.039352 | -1.902656 | -0.100795 |
| C | -2.138025 | 0.704876 | -0.053429 |
| C | -1.855121 | -0.690359 | 0.036134 |
| C | 0.090490 | 1.180690 | 0.066038 |
| C | 1.192489 | 2.144959 | 0.072080 |
| C | 2.468055 | 1.763657 | 0.189457 |
| C | 2.893601 | 0.336320 | 0.359214 |
| C | 1.774383 | -0.697636 | 0.274957 |
| C | 0.363741 | -0.221327 | 0.157894 |
| H | 0.916732 | 3.195333 | -0.029685 |
| H | 3.274368 | 2.499169 | 0.174876 |
| N | -0.579730 | -1.136022 | 0.140324 |
| N | -1.144480 | 1.631340 | -0.040048 |
| O | 2.046393 | -1.886722 | 0.317790 |
| H | 3.303569 | 0.217293 | 1.385782 |
| N | 3.995561 | 0.082709 | -0.588532 |
| O | 4.429118 | -1.187620 | -0.534075 |
| H | 3.749696 | -1.744748 | -0.081440 |


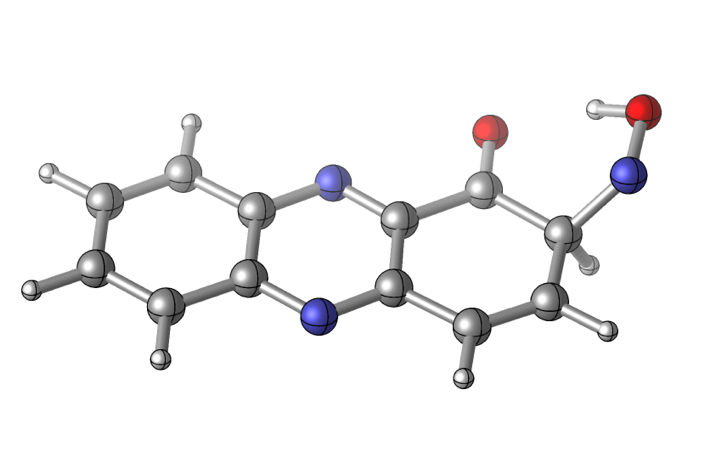


Zero-point correction= 0.183897 (Hartree/Particle)

Thermal correction to Energy= 0.196224

Thermal correction to Enthalpy= 0.197168

Thermal correction to Gibbs Free Energy= 0.143756

Sum of electronic and zero-point Energies= -776.309392

Sum of electronic and thermal Energies= -776.297066

Sum of electronic and thermal Enthalpies= -776.296122

Sum of electronic and thermal Free Energies= -776.349534

**^2^TS_3’-4_**

E(scf) = -906.3389236132 a.u.

ν_min_ = -1651.8 cm^-1^

| C | 0.305322 | -0.819161 | -0.228938 |
| --- | --- | --- | --- |
| C | 1.302171 | 0.104616 | -0.040007 |
| C | 1.915174 | -2.584471 | -0.646269 |
| C | 0.611894 | -2.174139 | -0.534500 |
| H | -0.739226 | -0.515495 | -0.144899 |
| H | 1.085606 | 1.147362 | 0.194762 |
| H | 2.179264 | -3.616540 | -0.879743 |
| H | -0.200818 | -2.886865 | -0.679981 |
| C | 2.662803 | -0.290091 | -0.148120 |
| C | 2.969592 | -1.649675 | -0.455288 |
| C | 4.888948 | 0.209518 | -0.064167 |
| C | 5.972104 | 1.170354 | 0.115946 |
| C | 7.255650 | 0.788125 | 0.062633 |
| C | 7.665586 | -0.627433 | -0.111248 |
| C | 6.604256 | -1.609608 | -0.505975 |
| C | 5.186325 | -1.157966 | -0.371781 |
| H | 5.688265 | 2.209688 | 0.282064 |
| H | 8.069021 | 1.505562 | 0.182641 |
| N | 4.252836 | -2.063923 | -0.568589 |
| N | 3.640326 | 0.630412 | 0.040145 |
| O | 6.881482 | -2.744710 | -0.877708 |
| H | 7.779738 | -1.023398 | 1.135374 |
| N | 8.956168 | -0.715662 | -0.623190 |
| O | 9.322647 | -1.938473 | -1.045901 |
| H | 8.532684 | -2.537022 | -1.014531 |

Zero-point correction= 0.185967 (Hartree/Particle)

Thermal correction to Energy= 0.200653

Thermal correction to Enthalpy= 0.201597

Thermal correction to Gibbs Free Energy= 0.143486

Sum of electronic and zero-point Energies= -906.152956

Sum of electronic and thermal Energies= -906.138271

Sum of electronic and thermal Enthalpies= -906.137327

Sum of electronic and thermal Free Energies= -906.195438

**^2^4**

E(scf) = -775.932953859 a.u.

| C | -4.485908 | 0.189889 | 0.000117 |
| --- | --- | --- | --- |
| C | -3.480996 | 1.123895 | 0.000006 |
| C | -2.892235 | -1.638600 | 0.000247 |
| C | -4.191494 | -1.202052 | 0.000237 |
| H | -5.527871 | 0.513453 | 0.000113 |
| H | -3.688300 | 2.194532 | -0.000089 |
| H | -2.637479 | -2.699017 | 0.000338 |
| H | -5.010888 | -1.921927 | 0.000322 |
| C | -2.123568 | 0.703349 | 0.000009 |
| C | -1.828935 | -0.693994 | 0.000134 |
| C | 0.106763 | 1.189880 | -0.000108 |
| C | 1.197010 | 2.158880 | -0.000247 |
| C | 2.482679 | 1.765458 | -0.000266 |
| C | 2.852665 | 0.356165 | -0.000138 |
| C | 1.803778 | -0.686257 | 0.000024 |
| C | 0.391051 | -0.214626 | 0.000025 |
| H | 0.919356 | 3.212889 | -0.000339 |
| H | 3.301802 | 2.485557 | -0.000373 |
| N | -0.550289 | -1.134581 | 0.000143 |
| N | -1.138646 | 1.634237 | -0.000111 |
| O | 2.087124 | -1.885276 | 0.000144 |
| N | 4.134431 | 0.091429 | -0.000171 |
| O | 4.517919 | -1.175419 | -0.000054 |
| H | 3.699688 | -1.749429 | 0.000048 |


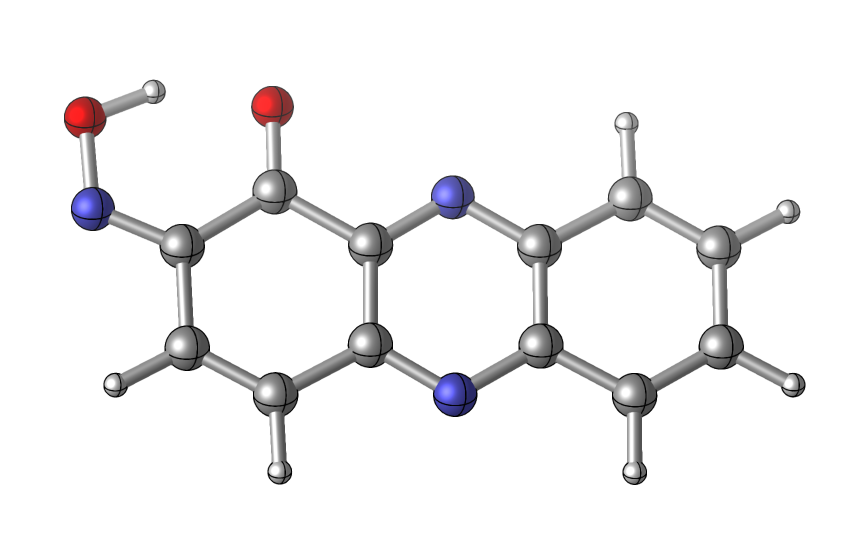


Zero-point correction= 0.174156 (Hartree/Particle)

Thermal correction to Energy= 0.185794

Thermal correction to Enthalpy= 0.186738

Thermal correction to Gibbs Free Energy= 0.135980

Sum of electronic and zero-point Energies= -775.758798

Sum of electronic and thermal Energies= -775.747160

Sum of electronic and thermal Enthalpies= -775.746216

Sum of electronic and thermal Free Energies= -775.796973

**^4^TS_2-3_**

E(scf) = -906.344243727 a.u.

ν_min_ = -603.0 cm^-1^

| C | -0.970555 | -0.043796 | 0.081656 |
| --- | --- | --- | --- |
| C | 0.256584 | -0.412204 | -0.406809 |
| C | -0.183914 | 2.203286 | 0.562505 |
| C | -1.191644 | 1.274121 | 0.571018 |
| H | -1.789534 | -0.764173 | 0.098908 |
| H | 0.449355 | -1.416987 | -0.784606 |
| H | -0.332372 | 3.219204 | 0.930505 |
| H | -2.176686 | 1.542592 | 0.955384 |
| C | 1.325133 | 0.525766 | -0.428208 |
| C | 1.099268 | 1.851259 | 0.059629 |
| C | 3.494704 | 1.044453 | -0.903575 |
| C | 4.844220 | 0.671436 | -1.348417 |
| C | 5.781203 | 1.736955 | -1.513127 |
| C | 5.534051 | 3.040222 | -1.061600 |
| C | 4.309941 | 3.369396 | -0.508450 |
| C | 3.259191 | 2.376140 | -0.438285 |
| H | 6.751114 | 1.504209 | -1.954227 |
| H | 6.303257 | 3.807946 | -1.147672 |
| N | 2.089340 | 2.769908 | 0.040907 |
| N | 2.535352 | 0.140059 | -0.901934 |
| O | 4.035279 | 4.595571 | -0.057897 |
| H | 3.108367 | 4.565211 | 0.250443 |
| H | 4.890089 | -0.175540 | -2.035734 |
| O | 6.528768 | -0.955736 | -0.135391 |
| N | 5.551793 | -0.290306 | 0.071620 |
| N | 5.768596 | 0.811912 | 1.277488 |
| O | 6.743870 | 0.543730 | 1.860960 |

Zero-point correction= 0.189159 (Hartree/Particle)

Thermal correction to Energy= 0.204039

Thermal correction to Enthalpy= 0.204983

Thermal correction to Gibbs Free Energy= 0.146159

Sum of electronic and zero-point Energies= -906.155085

Sum of electronic and thermal Energies= -906.140205

Sum of electronic and thermal Enthalpies= -906.139261

Sum of electronic and thermal Free Energies= -906.198084

**^4^2**

E(scf) = -906.364453747 a.u.

| C | -4.040079 | -1.546030 | -0.138133 |
| --- | --- | --- | --- |
| C | -2.734550 | -1.781168 | -0.508746 |
| C | -3.501418 | 0.730145 | 0.508707 |
| C | -4.423226 | -0.282460 | 0.374040 |
| H | -4.786326 | -2.335362 | -0.237675 |
| H | -2.414266 | -2.745945 | -0.904420 |
| H | -3.777828 | 1.710144 | 0.899956 |
| H | -5.461516 | -0.113374 | 0.663509 |
| C | -1.767250 | -0.758772 | -0.379055 |
| C | -2.151825 | 0.513528 | 0.133082 |
| C | 0.385563 | -0.042952 | -0.605026 |
| C | 1.832227 | -0.310121 | -0.976131 |
| C | 2.733730 | 0.885456 | -0.875946 |
| C | 2.307331 | 2.090718 | -0.396037 |
| C | 0.969000 | 2.295421 | 0.005360 |
| C | 0.001409 | 1.244667 | -0.103242 |
| H | 3.768903 | 0.743932 | -1.186840 |
| H | 3.000311 | 2.930391 | -0.324051 |
| N | -1.244429 | 1.517923 | 0.261536 |
| N | -0.471232 | -1.013074 | -0.747935 |
| O | 0.566710 | 3.478471 | 0.478190 |
| H | -0.386485 | 3.375471 | 0.670009 |
| H | 1.871510 | -0.762132 | -1.976633 |
| O | 2.941671 | -2.384438 | -0.534687 |
| N | 2.375219 | -1.385341 | -0.095329 |
| N | 2.263628 | -1.111108 | 1.288307 |
| O | 2.748861 | -1.980597 | 1.958604 |


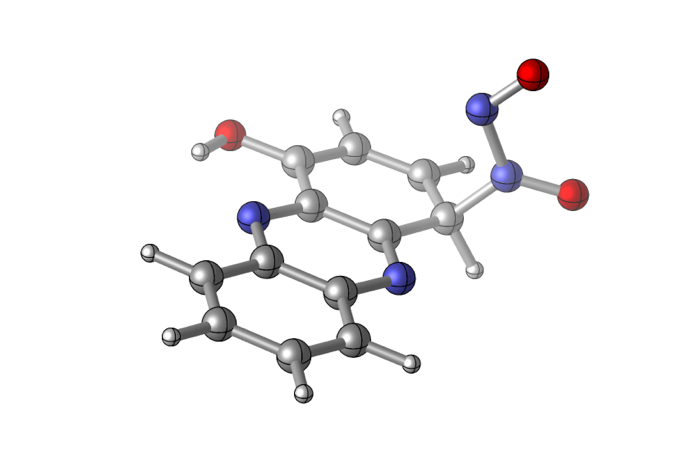


Zero-point correction= 0.191864 (Hartree/Particle)

Thermal correction to Energy= 0.206415

Thermal correction to Enthalpy= 0.207359

Thermal correction to Gibbs Free Energy= 0.148962

Sum of electronic and zero-point Energies= -906.172589

Sum of electronic and thermal Energies= -906.158039

Sum of electronic and thermal Enthalpies= -906.157095

Sum of electronic and thermal Free Energies= -906.215491

**^4^TS_2-3_**

E(scf) = -906.345764023 a.u.

ν_min_ = -274.9 cm^-1^

| C | -1.115227 | -0.023313 | -0.002229 |
| --- | --- | --- | --- |
| C | 0.158393 | -0.323038 | -0.433595 |
| C | -0.440844 | 2.234790 | 0.574428 |
| C | -1.414172 | 1.264386 | 0.505067 |
| H | -1.900126 | -0.779406 | -0.049749 |
| H | 0.414108 | -1.307919 | -0.827129 |
| H | -0.652057 | 3.232774 | 0.961201 |
| H | -2.427526 | 1.486339 | 0.843417 |
| C | 1.177457 | 0.654552 | -0.372313 |
| C | 0.877150 | 1.950835 | 0.135369 |
| C | 3.349707 | 1.263329 | -0.725200 |
| C | 4.755545 | 0.920046 | -1.142703 |
| C | 5.709480 | 2.067570 | -1.120430 |
| C | 5.371430 | 3.302379 | -0.640524 |
| C | 4.064538 | 3.579676 | -0.183700 |
| C | 3.046562 | 2.575340 | -0.224341 |
| H | 6.728024 | 1.862189 | -1.450852 |
| H | 6.111579 | 4.103433 | -0.604564 |
| N | 1.834281 | 2.914268 | 0.200643 |
| N | 2.440021 | 0.331807 | -0.802132 |
| O | 3.741772 | 4.789170 | 0.289270 |
| H | 2.796256 | 4.730558 | 0.530543 |
| H | 4.742754 | 0.414708 | -2.121313 |
| O | 6.321268 | -0.668789 | -0.503395 |
| N | 5.271264 | -0.165921 | -0.182609 |
| N | 5.545136 | 0.687709 | 1.681271 |
| O | 6.537945 | 0.238000 | 2.014883 |

Zero-point correction= 0.188809 (Hartree/Particle)

Thermal correction to Energy= 0.203860

Thermal correction to Enthalpy= 0.204805

Thermal correction to Gibbs Free Energy= 0.144846

Sum of electronic and zero-point Energies= -906.156955

Sum of electronic and thermal Energies= -906.141904

Sum of electronic and thermal Enthalpies= -906.140959

Sum of electronic and thermal Free Energies= -906.200918

**^4^3**

E(scf) = -776.489168375 a.u.

| C | -4.046293 | -0.759610 | -0.128235 |
| --- | --- | --- | --- |
| C | -2.859102 | -1.406026 | -0.385073 |
| C | -2.878281 | 1.317145 | 0.331869 |
| C | -4.053760 | 0.610747 | 0.234551 |
| H | -4.990088 | -1.301739 | -0.201141 |
| H | -2.827167 | -2.461020 | -0.660855 |
| H | -2.863444 | 2.372971 | 0.605785 |
| H | -5.004282 | 1.106934 | 0.436521 |
| C | -1.633128 | -0.704670 | -0.289531 |
| C | -1.641184 | 0.675331 | 0.066944 |
| C | 0.642134 | -0.682285 | -0.430159 |
| C | 1.958859 | -1.379616 | -0.542090 |
| C | 3.107847 | -0.489492 | -0.849059 |
| C | 3.057636 | 0.854566 | -0.556451 |
| C | 1.864024 | 1.459090 | -0.128694 |
| C | 0.630926 | 0.716657 | -0.116167 |
| H | 4.033660 | -0.942966 | -1.201518 |
| H | 3.940451 | 1.483844 | -0.680613 |
| N | -0.483274 | 1.382556 | 0.144105 |
| N | -0.462142 | -1.374246 | -0.524936 |
| O | 1.815110 | 2.758219 | 0.181995 |
| H | 0.874959 | 2.948685 | 0.370289 |
| H | 1.905297 | -2.275747 | -1.170253 |
| O | 1.907997 | -1.353847 | 1.792601 |
| N | 2.409371 | -1.927877 | 0.874772 |


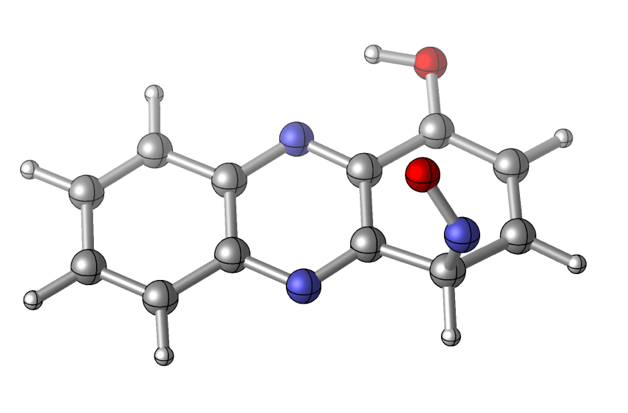


Zero-point correction= 0.182541 (Hartree/Particle)

Thermal correction to Energy= 0.195235

Thermal correction to Enthalpy= 0.196179

Thermal correction to Gibbs Free Energy= 0.142126

Sum of electronic and zero-point Energies= -776.306627

Sum of electronic and thermal Energies= -776.293933

Sum of electronic and thermal Enthalpies= -776.292989

Sum of electronic and thermal Free Energies= -776.347043

**^4^TS_3-3’_**

E(scf) = -852.888360721 a.u.

ν_min_ = -198.1 cm^-1^

| C | 0.041553 | -0.416108 | 0.012064 |
| --- | --- | --- | --- |
| C | 1.144419 | -1.230202 | 0.093497 |
| C | 1.410867 | 1.488587 | -0.600687 |
| C | 0.174758 | 0.954754 | -0.335149 |
| H | -0.950119 | -0.821419 | 0.218764 |
| H | 1.063522 | -2.283568 | 0.364959 |
| H | 1.540314 | 2.536704 | -0.873910 |
| H | -0.715426 | 1.582826 | -0.390718 |
| C | 2.436451 | -0.705399 | -0.172460 |
| C | 2.568078 | 0.667795 | -0.531249 |
| C | 4.684501 | -0.976441 | -0.343352 |
| C | 5.961675 | -1.689029 | -0.091270 |
| C | 6.997008 | -1.366607 | -1.091866 |
| C | 7.122465 | -0.102389 | -1.555567 |
| C | 6.166986 | 0.904968 | -1.114118 |
| C | 4.807733 | 0.386741 | -0.750455 |
| H | 7.749214 | -2.121066 | -1.328426 |
| H | 7.968618 | 0.223347 | -2.162247 |
| N | 3.784773 | 1.207628 | -0.811547 |
| N | 3.524368 | -1.516702 | -0.050735 |
| O | 6.399729 | 2.127983 | -1.096371 |
| O | 6.700773 | 0.163086 | 1.196145 |
| N | 6.414496 | -1.080915 | 1.324966 |
| H | 5.875866 | 2.511597 | 0.318398 |
| O | 5.722551 | 2.382463 | 1.330597 |
| H | 6.043755 | 1.365815 | 1.458737 |
| H | 4.764517 | 2.425126 | 1.479897 |
| H | 5.815343 | -2.756589 | 0.092369 |

Zero-point correction= 0.206294 (Hartree/Particle)

Thermal correction to Energy= 0.219785

Thermal correction to Enthalpy= 0.220730

Thermal correction to Gibbs Free Energy= 0.166102

Sum of electronic and zero-point Energies= -852.682067

Sum of electronic and thermal Energies= -852.668575

Sum of electronic and thermal Enthalpies= -852.667631

Sum of electronic and thermal Free Energies= -852.722259

**^4^3’**

E(scf) = -776.496791130 a.u.

| C | 3.939856 | 0.776187 | -0.073746 |
| --- | --- | --- | --- |
| C | 2.713138 | 1.375776 | -0.217731 |
| C | 2.926402 | -1.413646 | 0.181651 |
| C | 4.048486 | -0.626736 | 0.126063 |
| H | 4.847011 | 1.380675 | -0.113766 |
| H | 2.611593 | 2.450216 | -0.374012 |
| H | 2.981907 | -2.491793 | 0.335197 |
| H | 5.036410 | -1.075598 | 0.235830 |
| C | 1.539267 | 0.583260 | -0.163959 |
| C | 1.642024 | -0.822280 | 0.039668 |
| C | -0.743923 | 0.391314 | -0.239791 |
| C | -2.083529 | 1.072137 | -0.399879 |
| C | -3.251923 | 0.143673 | -0.274097 |
| C | -3.156268 | -1.178750 | -0.089342 |
| C | -1.863720 | -1.874758 | 0.044248 |
| C | -0.629593 | -1.012573 | -0.027751 |
| H | -4.232712 | 0.619644 | -0.342531 |
| H | -4.045718 | -1.806919 | -0.017770 |
| N | 0.537232 | -1.604684 | 0.102258 |
| N | 0.319618 | 1.162596 | -0.316187 |
| O | -1.784327 | -3.076709 | 0.212221 |
| H | -2.102076 | 1.502642 | -1.422926 |
| O | -1.319712 | 3.083056 | 0.521617 |
| N | -2.317559 | 2.185423 | 0.544159 |
| H | -0.520425 | 2.685405 | 0.085659 |


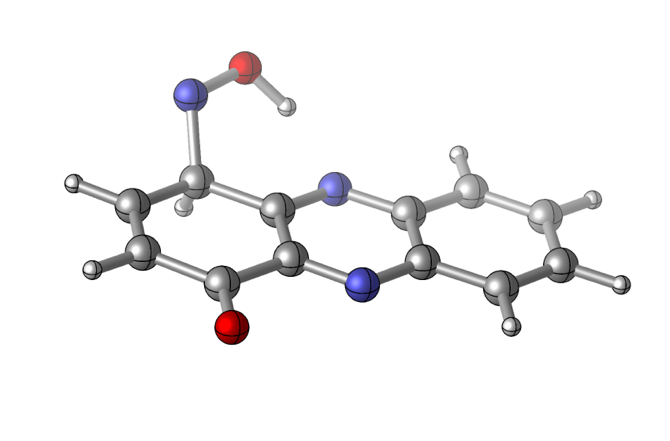


Zero-point correction= 0.184243 (Hartree/Particle)

Thermal correction to Energy= 0.196547

Thermal correction to Enthalpy= 0.197491

Thermal correction to Gibbs Free Energy= 0.144474

Sum of electronic and zero-point Energies= -776.312549

Sum of electronic and thermal Energies= -776.300244

Sum of electronic and thermal Enthalpies= -776.299300

Sum of electronic and thermal Free Energies= -776.352317

**^4^TS_3’-4_**

E(scf) = -906.335507352 a.u.

ν_min_ = -2059.3 cm^-1^

| C | -2.136858 | 2.465421 | -0.034217 |
| --- | --- | --- | --- |
| C | -3.384497 | 1.892953 | -0.053360 |
| C | -3.077008 | 4.702402 | -0.048475 |
| C | -1.980691 | 3.878135 | -0.030240 |
| H | -1.249512 | 1.831152 | -0.020229 |
| H | -3.522165 | 0.811223 | -0.054012 |
| H | -2.985002 | 5.788917 | -0.047535 |
| H | -0.977051 | 4.304506 | -0.012942 |
| C | -4.532296 | 2.724792 | -0.070902 |
| C | -4.381818 | 4.140672 | -0.072275 |
| C | -6.813386 | 2.980804 | -0.109685 |
| C | -8.178322 | 2.378789 | -0.072191 |
| C | -9.294403 | 3.321790 | -0.287332 |
| C | -9.156728 | 4.659339 | -0.291323 |
| C | -7.848456 | 5.314847 | -0.168542 |
| C | -6.649373 | 4.400990 | -0.119546 |
| H | -10.276664 | 2.860435 | -0.408379 |
| H | -10.019558 | 5.316626 | -0.406644 |
| N | -5.460561 | 4.960663 | -0.095899 |
| N | -5.771641 | 2.172634 | -0.072721 |
| O | -7.717080 | 6.525409 | -0.128514 |
| H | -8.240519 | 2.216580 | 1.263613 |
| O | -7.462193 | 0.244586 | -0.599541 |
| N | -8.470042 | 1.132213 | -0.609359 |
| N | -8.092480 | 2.229669 | 2.630106 |

Zero-point correction= 0.185116 (Hartree/Particle)

Thermal correction to Energy= 0.200161

Thermal correction to Enthalpy= 0.201105

Thermal correction to Gibbs Free Energy= 0.142050

Sum of electronic and zero-point Energies= -906.150392

Sum of electronic and thermal Energies= -906.135347

Sum of electronic and thermal Enthalpies= -906.134402

Sum of electronic and thermal Free Energies= -906.193458

**^4^4**

E(scf) = -775.936140550 a.u.

| C | 3.923677 | 0.785467 | 0.000112 |
| --- | --- | --- | --- |
| C | 2.694082 | 1.396317 | 0.000148 |
| C | 2.914090 | -1.421560 | -0.000139 |
| C | 4.035631 | -0.631020 | -0.000036 |
| H | 4.830302 | 1.391923 | 0.000197 |
| H | 2.589932 | 2.481755 | 0.000256 |
| H | 2.972837 | -2.510323 | -0.000237 |
| H | 5.025718 | -1.088322 | -0.000057 |
| C | 1.521570 | 0.599941 | 0.000024 |
| C | 1.627646 | -0.820406 | -0.000108 |
| C | -0.766911 | 0.411472 | -0.000150 |
| C | -2.100542 | 1.028574 | -0.000103 |
| C | -3.263602 | 0.150702 | -0.000041 |
| C | -3.170899 | -1.192517 | -0.000055 |
| C | -1.877370 | -1.888988 | 0.000041 |
| C | -0.646577 | -1.013909 | -0.000119 |
| H | -4.234536 | 0.649182 | -0.000027 |
| H | -4.061046 | -1.822476 | -0.000039 |
| N | 0.523880 | -1.608801 | -0.000129 |
| N | 0.299976 | 1.190380 | 0.000041 |
| O | -1.781765 | -3.103272 | 0.000326 |
| N | -2.368108 | 2.304546 | 0.000015 |
| O | -1.363258 | 3.177553 | 0.000026 |
| H | -0.498005 | 2.676704 | 0.000169 |


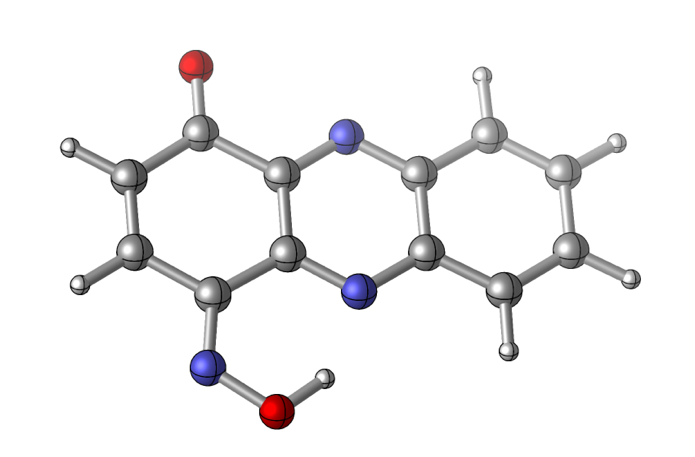


Zero-point correction= 0.174218 (Hartree/Particle)

Thermal correction to Energy= 0.185949

Thermal correction to Enthalpy= 0.186894

Thermal correction to Gibbs Free Energy= 0.135976

Sum of electronic and zero-point Energies= -775.761922

Sum of electronic and thermal Energies= -775.750191

Sum of electronic and thermal Enthalpies= -775.749247

Sum of electronic and thermal Free Energies= -775.800165

**^2^Int_I_**

E(scf) = -776.474903506 a.u.

| C | -4.459115 | -0.487149 | 0.243972 |
| --- | --- | --- | --- |
| C | -3.357613 | -1.312553 | 0.141412 |
| C | -3.047279 | 1.479329 | 0.112156 |
| C | -4.299679 | 0.918494 | 0.229146 |
| H | -5.457436 | -0.917229 | 0.337191 |
| H | -3.454467 | -2.399524 | 0.150838 |
| H | -2.902584 | 2.560826 | 0.098514 |
| H | -5.178027 | 1.560916 | 0.311251 |
| C | -2.063973 | -0.761766 | 0.020712 |
| C | -1.897153 | 0.653409 | 0.004529 |
| C | 0.192994 | -1.050269 | -0.191842 |
| C | 1.368581 | -1.917047 | -0.287764 |
| C | 2.606307 | -1.425494 | -0.417596 |
| C | 2.874070 | 0.039327 | -0.481947 |
| C | 1.669829 | 0.911837 | -0.340160 |
| C | 0.369255 | 0.393229 | -0.210416 |
| H | 1.182569 | -2.991651 | -0.251129 |
| H | 3.469702 | -2.087924 | -0.479972 |
| N | -0.674720 | 1.232089 | -0.109762 |
| N | -0.983544 | -1.606075 | -0.078011 |
| N | 3.888984 | 0.557945 | 0.546408 |
| O | 4.507529 | -0.288583 | 1.107787 |
| H | 3.398448 | 0.299922 | -1.425726 |
| O | 1.842702 | 2.238588 | -0.347880 |


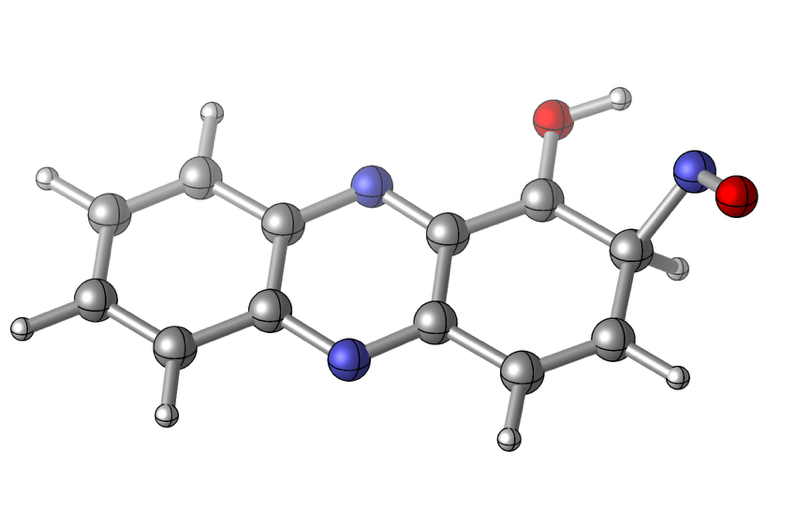


Zero-point correction= 0.182600 (Hartree/Particle)

Thermal correction to Energy= 0.195451

Thermal correction to Enthalpy= 0.196395

Thermal correction to Gibbs Free Energy= 0.141748

Sum of electronic and zero-point Energies= -776.292304

Sum of electronic and thermal Energies= -776.279453

Sum of electronic and thermal Enthalpies= -776.278509

Sum of electronic and thermal Free Energies= -776.333155

**^2^TS_I_**

E(scf) = -906.321865461 a.u.

ν_min_ = -1879.8cm^-1^

| C | -0.634628 | -0.631292 | 0.199388 |
| --- | --- | --- | --- |
| C | -1.440493 | 0.484208 | 0.186085 |
| C | -2.466687 | -2.031191 | -0.551719 |
| C | -1.151748 | -1.897711 | -0.171923 |
| H | 0.411308 | -0.544571 | 0.497291 |
| H | -1.063902 | 1.468647 | 0.467612 |
| H | -2.887023 | -2.995171 | -0.841912 |
| H | -0.497838 | -2.770867 | -0.155111 |
| C | -2.798571 | 0.373678 | -0.198721 |
| C | -3.318673 | -0.897322 | -0.572639 |
| C | -4.836982 | 1.333834 | -0.562999 |
| C | -5.719829 | 2.501459 | -0.593458 |
| C | -7.011569 | 2.411096 | -0.932832 |
| C | -7.670579 | 1.117500 | -1.279044 |
| C | -6.764190 | -0.080113 | -1.303067 |
| C | -5.361586 | 0.045005 | -0.939251 |
| H | -5.265232 | 3.458781 | -0.333190 |
| H | -7.640670 | 3.301704 | -0.942152 |
| N | -4.617673 | -1.045768 | -0.949527 |
| N | -3.584436 | 1.491758 | -0.206113 |
| N | -8.832126 | 0.746150 | -0.332661 |
| O | -9.128891 | 1.606633 | 0.433774 |
| H | -8.196248 | 1.178918 | -2.249219 |
| O | -7.220281 | -1.192451 | -1.769884 |

Zero-point correction= 0.184762 (Hartree/Particle)

Thermal correction to Energy= 0.199745

Thermal correction to Enthalpy= 0.200689

Thermal correction to Gibbs Free Energy= 0.141467

Sum of electronic and zero-point Energies= -906.137103

Sum of electronic and thermal Energies= -906.122120

Sum of electronic and thermal Enthalpies= -906.121176

Sum of electronic and thermal Free Energies= -906.180398

**^2^INT_II_**

E(scf) = -775.893271057 a.u.

| C | -4.412941 | -0.515433 | 0.270029 |
| --- | --- | --- | --- |
| C | -3.316845 | -1.334215 | 0.150331 |
| C | -3.032577 | 1.473418 | 0.128503 |
| C | -4.271655 | 0.898195 | 0.259291 |
| H | -5.407548 | -0.951351 | 0.375046 |
| H | -3.405505 | -2.421182 | 0.156429 |
| H | -2.895437 | 2.555340 | 0.116984 |
| H | -5.158585 | 1.525663 | 0.356028 |
| C | -2.023010 | -0.767350 | 0.014263 |
| C | -1.881692 | 0.650634 | 0.003340 |
| C | 0.233313 | -1.006392 | -0.225906 |
| C | 1.419687 | -1.857188 | -0.350896 |
| C | 2.652383 | -1.350757 | -0.463265 |
| C | 2.922986 | 0.115035 | -0.473067 |
| C | 1.721705 | 1.051684 | -0.380807 |
| C | 0.367175 | 0.419636 | -0.236033 |
| H | 1.242124 | -2.933655 | -0.349264 |
| H | 3.515490 | -2.012359 | -0.543705 |
| N | -0.660957 | 1.228710 | -0.125697 |
| N | -0.944259 | -1.585895 | -0.103229 |
| O | 1.874485 | 2.251310 | -0.411365 |
| N | 3.900744 | 0.584739 | 0.612506 |
| O | 4.413135 | -0.305264 | 1.213352 |
| H | 3.468621 | 0.412698 | -1.387177 |


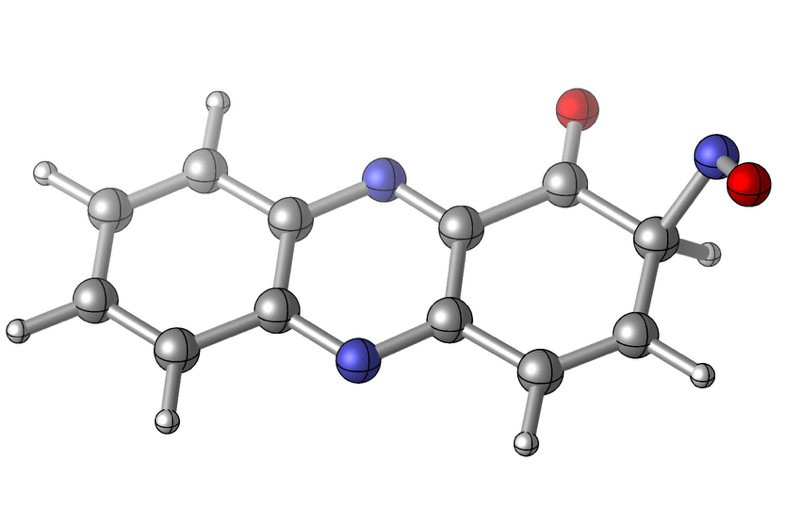


Zero-point correction= 0.171862 (Hartree/Particle)

Thermal correction to Energy= 0.184270

Thermal correction to Enthalpy= 0.185214

Thermal correction to Gibbs Free Energy= 0.131822

Sum of electronic and zero-point Energies= -775.721409

Sum of electronic and thermal Energies= -775.709001

Sum of electronic and thermal Enthalpies= -775.708057

Sum of electronic and thermal Free Energies= -775.761449

**^2^TS_II_**

E(scf) = -852.286628227 a.u.

ν_min_ = -103.7 cm^-1^

| C | -1.672812 | -1.882186 | -0.110363 |
| --- | --- | --- | --- |
| C | -0.585747 | -2.677395 | 0.151576 |
| C | -0.288060 | 0.107238 | -0.177919 |
| C | -1.524804 | -0.477556 | -0.276751 |
| H | -2.665462 | -2.327725 | -0.193167 |
| H | -0.679159 | -3.756421 | 0.282046 |
| H | -0.145888 | 1.181858 | -0.300825 |
| H | -2.404700 | 0.133105 | -0.484063 |
| C | 0.709339 | -2.099215 | 0.258382 |
| C | 0.856810 | -0.691953 | 0.091918 |
| C | 2.960201 | -2.305599 | 0.596672 |
| C | 4.128998 | -3.119943 | 0.879384 |
| C | 5.356557 | -2.562200 | 0.926547 |
| C | 5.583055 | -1.145189 | 0.679240 |
| C | 4.454525 | -0.215054 | 0.540702 |
| C | 3.103152 | -0.882895 | 0.429023 |
| H | 3.968181 | -4.182845 | 1.061410 |
| H | 6.242296 | -3.159977 | 1.147391 |
| N | 2.072750 | -0.101173 | 0.187726 |
| N | 1.775831 | -2.894256 | 0.515849 |
| O | 4.557722 | 1.002316 | 0.477099 |
| N | 6.827466 | -0.562774 | 0.888369 |
| O | 7.785619 | -1.342951 | 0.971296 |
| H | 6.065856 | -1.382650 | -0.888195 |

Zero-point correction= 0.195228 (Hartree/Particle)

Thermal correction to Energy= 0.209597

Thermal correction to Enthalpy= 0.210541

Thermal correction to Gibbs Free Energy= 0.153745

Sum of electronic and zero-point Energies= -852.091400

Sum of electronic and thermal Energies= -852.077031

Sum of electronic and thermal Enthalpies= -852.076087

Sum of electronic and thermal Free Energies= -852.132884

**^4^INT_I_**

E(scf) = -776.480081245 a.u.

| C | -4.065649 | -0.958615 | -0.071913 |
| --- | --- | --- | --- |
| C | -2.832372 | -1.515539 | -0.327921 |
| C | -3.052512 | 1.200071 | 0.373916 |
| C | -4.174078 | 0.408823 | 0.283093 |
| H | -4.966356 | -1.570387 | -0.140512 |
| H | -2.721823 | -2.566475 | -0.599582 |
| H | -3.114305 | 2.255725 | 0.642525 |
| H | -5.158342 | 0.834826 | 0.484261 |
| C | -1.664653 | -0.722834 | -0.237120 |
| C | -1.769405 | 0.651583 | 0.111641 |
| C | 0.604535 | -0.523510 | -0.383381 |
| C | 1.956768 | -1.165398 | -0.556136 |
| C | 3.090012 | -0.216745 | -0.687578 |
| C | 2.945010 | 1.110024 | -0.404773 |
| C | 1.694779 | 1.677914 | -0.052874 |
| C | 0.499763 | 0.880822 | -0.060915 |
| H | 4.067477 | -0.615779 | -0.955753 |
| H | 3.813035 | 1.772378 | -0.463696 |
| N | -0.673865 | 1.451590 | 0.188714 |
| N | -0.444295 | -1.294555 | -0.480564 |
| N | 2.101185 | -2.145635 | 0.646104 |
| O | 3.139106 | -2.061627 | 1.219367 |
| H | 1.900946 | -1.885149 | -1.389900 |
| O | 1.547976 | 2.976522 | 0.256418 |


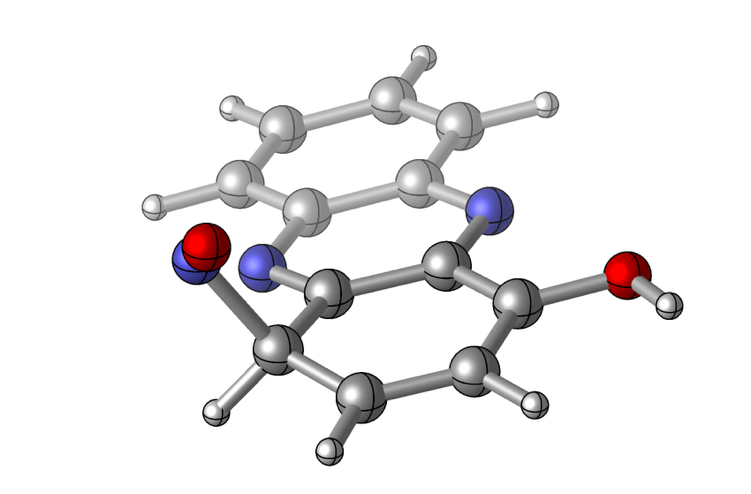


Zero-point correction= 0.182368 (Hartree/Particle)

Thermal correction to Energy= 0.195201

Thermal correction to Enthalpy= 0.196145

Thermal correction to Gibbs Free Energy= 0.141562

Sum of electronic and zero-point Energies= -776.297713

Sum of electronic and thermal Energies= -776.284880

Sum of electronic and thermal Enthalpies= -776.283936

Sum of electronic and thermal Free Energies= -776.338519

**^4^TS_I_**

E(scf) = -906.323952492 a.u.

ν_min_ = -1952.0 cm^-1^

| C | -0.352306 | 0.032038 | 0.023783 |
| --- | --- | --- | --- |
| C | 0.768730 | -0.741495 | -0.163267 |
| C | 0.947126 | 2.070550 | -0.182800 |
| C | -0.261540 | 1.447971 | 0.015515 |
| H | -1.322112 | -0.442189 | 0.181214 |
| H | 0.723955 | -1.831392 | -0.157947 |
| H | 1.040236 | 3.157314 | -0.196335 |
| H | -1.163005 | 2.043532 | 0.167233 |
| C | 2.028049 | -0.123824 | -0.364503 |
| C | 2.119909 | 1.295374 | -0.380242 |
| C | 4.275593 | -0.280849 | -0.711684 |
| C | 5.520421 | -1.124041 | -0.820711 |
| C | 6.753465 | -0.392536 | -1.224921 |
| C | 6.814793 | 0.952114 | -1.269774 |
| C | 5.664344 | 1.788448 | -0.998229 |
| C | 4.364288 | 1.150949 | -0.755226 |
| H | 7.639759 | -0.989255 | -1.442092 |
| H | 7.738606 | 1.467770 | -1.537774 |
| N | 3.307622 | 1.921840 | -0.589912 |
| N | 3.136407 | -0.901077 | -0.534624 |
| N | 5.655035 | -1.842087 | 0.543499 |
| O | 6.740769 | -1.770242 | 1.024665 |
| H | 5.306025 | -1.978685 | -1.484272 |
| O | 5.733796 | 3.072852 | -1.137769 |

Zero-point correction= 0.184468 (Hartree/Particle)

Thermal correction to Energy= 0.199596

Thermal correction to Enthalpy= 0.200540

Thermal correction to Gibbs Free Energy= 0.140464

Sum of electronic and zero-point Energies= -906.139484

Sum of electronic and thermal Energies= -906.124357

Sum of electronic and thermal Enthalpies= -906.123413

Sum of electronic and thermal Free Energies= -906.183489

**^4^INT_II_**

E(scf) = -775.894727217 a.u.

| C | -4.019066 | -1.027035 | -0.052834 |
| --- | --- | --- | --- |
| C | -2.774714 | -1.562291 | -0.273925 |
| C | -3.083606 | 1.180998 | 0.318432 |
| C | -4.175455 | 0.353995 | 0.246140 |
| H | -4.902721 | -1.664600 | -0.105976 |
| H | -2.632967 | -2.618858 | -0.503909 |
| H | -3.176871 | 2.243922 | 0.544095 |
| H | -5.175768 | 0.753766 | 0.417513 |
| C | -1.626751 | -0.730289 | -0.202145 |
| C | -1.782423 | 0.654316 | 0.094366 |
| C | 0.628418 | -0.449037 | -0.337205 |
| C | 1.997629 | -1.040072 | -0.542390 |
| C | 3.126489 | -0.067185 | -0.544275 |
| C | 2.978030 | 1.240996 | -0.301365 |
| C | 1.668056 | 1.853877 | -0.006039 |
| C | 0.471568 | 0.938806 | -0.050084 |
| H | 4.119772 | -0.476774 | -0.734486 |
| H | 3.832114 | 1.920444 | -0.303517 |
| N | -0.709249 | 1.479216 | 0.158866 |
| N | -0.397563 | -1.265410 | -0.419892 |
| O | 1.551798 | 3.035878 | 0.255551 |
| N | 2.158758 | -2.159052 | 0.498641 |
| O | 3.215911 | -2.161137 | 1.045950 |
| H | 1.982080 | -1.621583 | -1.481075 |


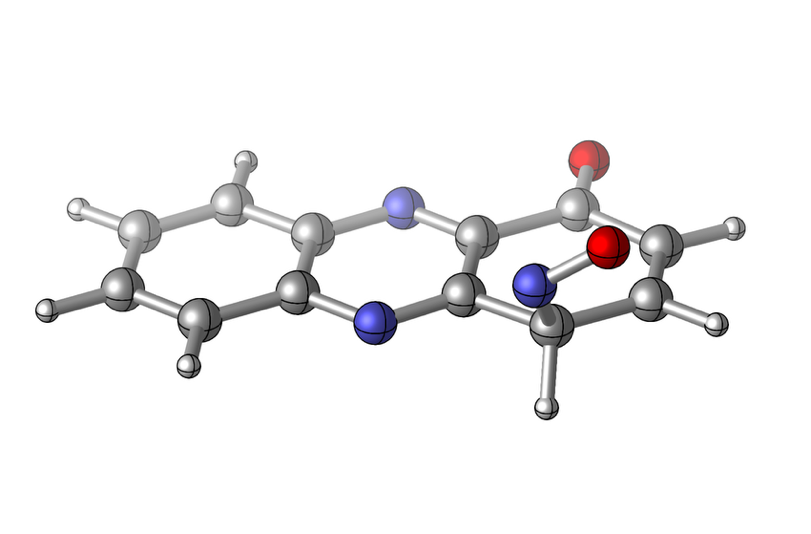


Zero-point correction= 0.172048 (Hartree/Particle)

Thermal correction to Energy= 0.184499

Thermal correction to Enthalpy= 0.185443

Thermal correction to Gibbs Free Energy= 0.131980

Sum of electronic and zero-point Energies= -775.722679

Sum of electronic and thermal Energies= -775.710228

Sum of electronic and thermal Enthalpies= -775.709284

Sum of electronic and thermal Free Energies= -775.762747

**^4^TS_II_**

E(scf) = -852.284484010 a.u.

ν_min_ = -120.4 cm^-1^

| C | -1.273977 | -0.515190 | -0.046988 |
| --- | --- | --- | --- |
| C | 0.043008 | -0.834368 | 0.165093 |
| C | -0.739424 | 1.838415 | -0.277961 |
| C | -1.671098 | 0.832745 | -0.271055 |
| H | -2.031112 | -1.301088 | -0.045493 |
| H | 0.366205 | -1.861922 | 0.337596 |
| H | -1.015242 | 2.880415 | -0.446609 |
| H | -2.725000 | 1.060192 | -0.437470 |
| C | 1.032975 | 0.188108 | 0.164358 |
| C | 0.634075 | 1.537171 | -0.058839 |
| C | 3.209653 | 0.847624 | 0.370116 |
| C | 4.635034 | 0.563230 | 0.556117 |
| C | 5.551576 | 1.677118 | 0.646873 |
| C | 5.184402 | 2.965651 | 0.445814 |
| C | 3.804500 | 3.338019 | 0.155893 |
| C | 2.803298 | 2.210397 | 0.147230 |
| H | 6.585455 | 1.422291 | 0.888646 |
| H | 5.909362 | 3.778092 | 0.517008 |
| N | 1.544989 | 2.537716 | -0.065862 |
| N | 2.329359 | -0.141291 | 0.367341 |
| O | 3.454374 | 4.493970 | -0.050520 |
| N | 4.986228 | -0.705965 | 0.997317 |
| O | 6.199807 | -0.924809 | 1.117166 |
| H | 5.158761 | 0.108802 | -0.923188 |

Zero-point correction= 0.194771 (Hartree/Particle)

Thermal correction to Energy= 0.209242

Thermal correction to Enthalpy= 0.210186

Thermal correction to Gibbs Free Energy= 0.153114

Sum of electronic and zero-point Energies= -852.089713

Sum of electronic and thermal Energies= -852.075242

Sum of electronic and thermal Enthalpies= -852.074298

Sum of electronic and thermal Free Energies= -852.131370

**^2^TS_2-2’_**

E(scf) = -982.785172750 a.u.

ν_min_ = -171.3 cm^-1^

| C | -1.540838 | 1.707710 | 0.286143 |
| --- | --- | --- | --- |
| C | -0.644781 | 0.690595 | 0.065234 |
| C | 0.198354 | 3.385339 | 0.079015 |
| C | -1.118345 | 3.064255 | 0.293005 |
| H | -2.592887 | 1.476245 | 0.458740 |
| H | -0.951077 | -0.355921 | 0.055307 |
| H | 0.549923 | 4.417647 | 0.079738 |
| H | -1.851286 | 3.852133 | 0.470733 |
| C | 0.723241 | 0.991536 | -0.158478 |
| C | 1.146937 | 2.353523 | -0.150206 |
| C | 2.861226 | 0.308361 | -0.572807 |
| C | 3.836894 | -0.755500 | -0.824724 |
| C | 5.134335 | -0.502861 | -1.025839 |
| C | 5.698226 | 0.882843 | -0.984629 |
| C | 4.696935 | 1.992384 | -0.771086 |
| C | 3.274641 | 1.679092 | -0.551138 |
| H | 3.445765 | -1.773419 | -0.840186 |
| H | 5.846268 | -1.309710 | -1.204458 |
| N | 2.446856 | 2.678820 | -0.354990 |
| N | 1.601298 | -0.022441 | -0.380049 |
| O | 4.994922 | 3.203335 | -1.084966 |
| H | 6.330822 | 1.092224 | -1.857389 |
| N | 6.525649 | 1.119827 | 0.232737 |
| O | 7.798359 | 1.095850 | 0.285892 |

Zero-point correction= 0.218554 (Hartree/Particle)

Thermal correction to Energy= 0.234248

Thermal correction to Enthalpy= 0.235193

Thermal correction to Gibbs Free Energy= 0.174908

Sum of electronic and zero-point Energies= -982.566619

Sum of electronic and thermal Energies= -982.550924

Sum of electronic and thermal Enthalpies= -982.549980

Sum of electronic and thermal Free Energies= -982.610264

**^2^2’**

E(scf) = -906.401218810 a.u.

| C | 5.070767 | 0.164540 | 0.271800 |
| --- | --- | --- | --- |
| C | 4.068146 | 1.101032 | 0.213966 |
| C | 3.484058 | -1.656402 | 0.055368 |
| C | 4.778736 | -1.223906 | 0.192691 |
| H | 6.107769 | 0.485650 | 0.380405 |
| H | 4.273583 | 2.170381 | 0.273420 |
| H | 3.231168 | -2.715414 | -0.006953 |
| H | 5.594402 | -1.946248 | 0.242742 |
| C | 2.719167 | 0.684073 | 0.071211 |
| C | 2.426918 | -0.709835 | -0.008031 |
| C | 0.501353 | 1.178383 | -0.130911 |
| C | -0.594556 | 2.151016 | -0.179057 |
| C | -1.857921 | 1.789542 | -0.424697 |
| C | -2.226749 | 0.372506 | -0.753976 |
| C | -1.200366 | -0.675973 | -0.314559 |
| C | 0.219748 | -0.223361 | -0.211807 |
| H | -0.325305 | 3.194819 | -0.014890 |
| H | -2.660506 | 2.526380 | -0.471501 |
| N | 1.149804 | -1.147269 | -0.138181 |
| N | 1.734559 | 1.619368 | 0.018278 |
| O | -1.547536 | -1.812607 | -0.068154 |
| H | -2.288874 | 0.289304 | -1.854833 |
| N | -3.547660 | 0.009765 | -0.261619 |
| O | -4.121629 | -1.095331 | -0.829961 |


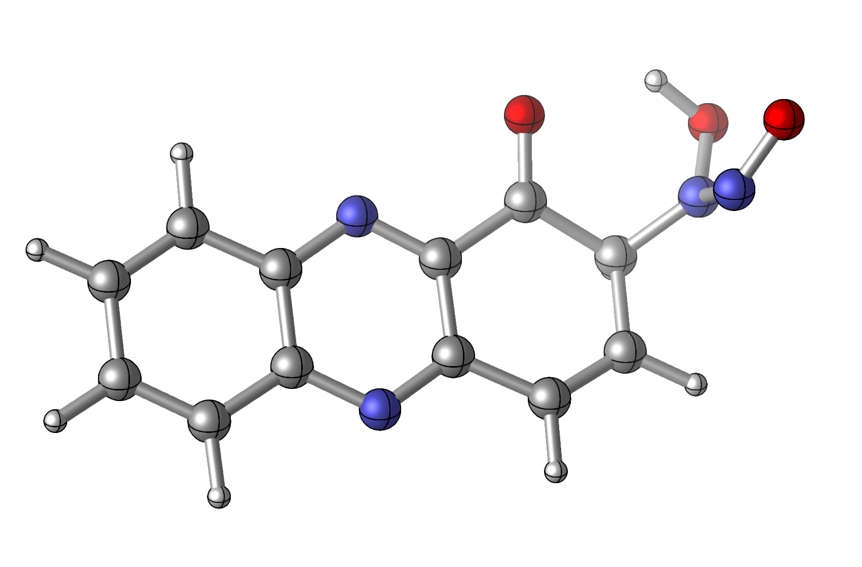


Zero-point correction= 0.193871 (Hartree/Particle)

Thermal correction to Energy= 0.208162

Thermal correction to Enthalpy= 0.209106

Thermal correction to Gibbs Free Energy= 0.151701

Sum of electronic and zero-point Energies= -906.207348

Sum of electronic and thermal Energies= -906.193057

Sum of electronic and thermal Enthalpies= -906.192113

Sum of electronic and thermal Free Energies= -906.249518

**^2^TS_2’-3*_**

E(scf) = -982.788519057 a.u.

ν_min_ = -395.3 cm^-1^

| C | -0.622591 | 0.262453 | 0.070185 |
| --- | --- | --- | --- |
| C | -1.595516 | 1.221904 | 0.167548 |
| C | -2.263078 | -1.520132 | 0.197099 |
| C | -0.957454 | -1.122628 | 0.084894 |
| H | 0.424470 | 0.556232 | -0.019914 |
| H | -1.358578 | 2.286695 | 0.157858 |
| H | -2.547623 | -2.573205 | 0.211743 |
| H | -0.162789 | -1.865888 | 0.006350 |
| C | -2.964537 | 0.841835 | 0.284815 |
| C | -3.298583 | -0.546724 | 0.300750 |
| C | -5.176207 | 1.392207 | 0.481117 |
| C | -6.221924 | 2.381025 | 0.601312 |
| C | -7.518474 | 1.987799 | 0.658513 |
| C | -7.891006 | 0.598408 | 0.504509 |
| C | -6.920094 | -0.450366 | 0.648495 |
| C | -5.503478 | -0.011658 | 0.495327 |
| H | -5.932950 | 3.430448 | 0.642252 |
| H | -8.322580 | 2.721097 | 0.748862 |
| N | -4.580818 | -0.952890 | 0.415956 |
| N | -3.912334 | 1.797320 | 0.380180 |
| O | -7.232587 | -1.631987 | 0.890664 |
| H | -7.995532 | 0.669633 | -1.139496 |
| N | -9.231324 | 0.181397 | 0.816956 |
| O | -9.429283 | -0.581563 | 1.935615 |

Zero-point correction= 0.216389 (Hartree/Particle)

Thermal correction to Energy= 0.232266

Thermal correction to Enthalpy= 0.233210

Thermal correction to Gibbs Free Energy= 0.172749

Sum of electronic and zero-point Energies= -982.572130

Sum of electronic and thermal Energies= -982.556253

Sum of electronic and thermal Enthalpies= -982.555309

Sum of electronic and thermal Free Energies= -982.615770

**^2^3***

E(scf) = -906.366237579 a.u.

| C | 5.182693 | 0.279922 | 0.071369 |
| --- | --- | --- | --- |
| C | 4.154509 | 1.182779 | -0.003691 |
| C | 3.635726 | -1.587246 | 0.127523 |
| C | 4.923210 | -1.119136 | 0.138033 |
| H | 6.215867 | 0.631171 | 0.080489 |
| H | 4.333953 | 2.257632 | -0.055302 |
| H | 3.407952 | -2.653049 | 0.177265 |
| H | 5.759933 | -1.816646 | 0.197527 |
| C | 2.803841 | 0.727782 | -0.015790 |
| C | 2.545206 | -0.674252 | 0.049940 |
| C | 0.557058 | 1.149640 | -0.095816 |
| C | -0.545492 | 2.072651 | -0.182684 |
| C | -1.821818 | 1.606979 | -0.198800 |
| C | -2.106907 | 0.208728 | -0.127143 |
| C | -1.094295 | -0.817536 | -0.033165 |
| C | 0.303470 | -0.267614 | -0.034357 |
| H | -0.323745 | 3.135069 | -0.271442 |
| H | -2.649034 | 2.312596 | -0.310663 |
| N | 1.281023 | -1.150561 | 0.038888 |
| N | 1.799731 | 1.626782 | -0.088562 |
| O | -1.316853 | -2.023722 | 0.068746 |
| N | -3.435335 | -0.192058 | -0.072486 |
| O | -3.885229 | -1.222097 | -0.789218 |
| N | -4.360454 | 0.404949 | 0.606986 |


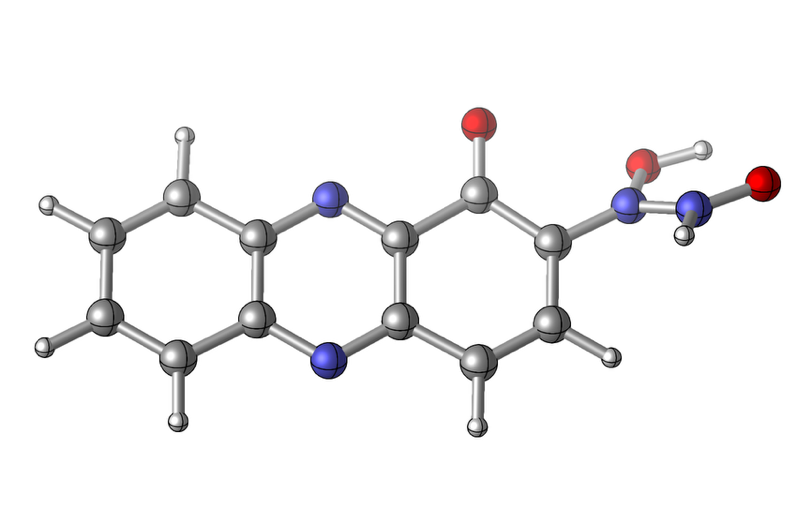


Zero-point correction= 0.194521 (Hartree/Particle)

Thermal correction to Energy= 0.208643

Thermal correction to Enthalpy= 0.209587

Thermal correction to Gibbs Free Energy= 0.152643

Sum of electronic and zero-point Energies= -906.171717

Sum of electronic and thermal Energies= -906.157595

Sum of electronic and thermal Enthalpies= -906.156651

Sum of electronic and thermal Free Energies= -906.213594

**^2^TS_3*-4’_**

E(scf) = -906.353450848 a.u.

ν_min_ = -544.5 cm^-1^

| C | -1.711142 | 1.669053 | 0.004692 |
| --- | --- | --- | --- |
| C | -0.692779 | 2.582689 | -0.072501 |
| C | -0.154490 | -0.190329 | -0.089784 |
| C | -1.441420 | 0.270303 | -0.004252 |
| H | -2.744847 | 2.011235 | 0.073802 |
| H | -0.879400 | 3.657223 | -0.067389 |
| H | 0.079689 | -1.255517 | -0.098529 |
| H | -2.271909 | -0.434171 | 0.057737 |
| C | 0.656392 | 2.137090 | -0.163231 |
| C | 0.926481 | 0.733331 | -0.171248 |
| C | 2.890272 | 2.578899 | -0.323738 |
| C | 3.989766 | 3.518706 | -0.416233 |
| C | 5.266372 | 3.084425 | -0.517464 |
| C | 5.581482 | 1.678761 | -0.524451 |
| C | 4.546743 | 0.674281 | -0.413360 |
| C | 3.151778 | 1.168741 | -0.330656 |
| H | 3.744338 | 4.580302 | -0.418134 |
| H | 6.084746 | 3.798948 | -0.615591 |
| N | 2.190531 | 0.267452 | -0.255563 |
| N | 1.651823 | 3.047236 | -0.240834 |
| O | 4.809369 | -0.547953 | -0.376703 |
| N | 6.879354 | 1.322335 | -0.628966 |
| O | 7.178584 | 0.041304 | -0.697651 |
| N | 8.052145 | 2.001219 | 0.563022 |

Zero-point correction= 0.190274 (Hartree/Particle)

Thermal correction to Energy= 0.204344

Thermal correction to Enthalpy= 0.205288

Thermal correction to Gibbs Free Energy= 0.148539

Sum of electronic and zero-point Energies= -906.163177

Sum of electronic and thermal Energies= -906.149107

Sum of electronic and thermal Enthalpies= -906.148163

Sum of electronic and thermal Free Energies= -906.204911

**^4^TS_2-2’_**

E(scf) = -982.786817815 a.u.

ν_min_ = -761.9 cm^-1^

| C | -0.292538 | -0.201623 | 0.114567 |
| --- | --- | --- | --- |
| C | -1.470143 | -0.747007 | 0.569094 |
| C | -1.414708 | 1.851736 | -0.521514 |
| C | -0.264591 | 1.104275 | -0.436534 |
| H | 0.631830 | -0.777816 | 0.173732 |
| H | -1.514503 | -1.752049 | 0.990101 |
| H | -1.418094 | 2.860002 | -0.937187 |
| H | 0.680345 | 1.515555 | -0.793925 |
| C | -2.670564 | -0.000446 | 0.490736 |
| C | -2.640128 | 1.315624 | -0.053722 |
| C | -4.899376 | 0.185697 | 0.837448 |
| C | -6.280251 | -0.318925 | 1.139413 |
| C | -7.081494 | 0.713843 | 1.890562 |
| C | -7.042872 | 1.990791 | 1.467811 |
| C | -6.135375 | 2.302699 | 0.366809 |
| C | -4.858650 | 1.516648 | 0.333993 |
| H | -7.790922 | 0.378841 | 2.647721 |
| H | -7.715574 | 2.768949 | 1.828915 |
| N | -3.772832 | 2.075133 | -0.131432 |
| N | -3.841234 | -0.571579 | 0.913250 |
| O | -6.302587 | 3.283915 | -0.399093 |
| O | -6.953250 | 0.603695 | -0.962198 |
| N | -6.954082 | -0.460520 | -0.186310 |
| N | -7.874188 | -1.412044 | -0.246904 |

Zero-point correction= 0.215453 (Hartree/Particle)

Thermal correction to Energy= 0.230840

Thermal correction to Enthalpy= 0.231785

Thermal correction to Gibbs Free Energy= 0.172806

Sum of electronic and zero-point Energies= -982.571364

Sum of electronic and thermal Energies= -982.555977

Sum of electronic and thermal Enthalpies= -982.555033

Sum of electronic and thermal Free Energies= -982.614012

**^4^2’**

E(scf) = -906.404990314 a.u.

| C | -3.889914 | -1.752253 | -0.046704 |
| --- | --- | --- | --- |
| C | -2.553628 | -1.901314 | -0.321194 |
| C | -3.613595 | 0.620012 | 0.386187 |
| C | -4.424312 | -0.483284 | 0.308663 |
| H | -4.555592 | -2.614806 | -0.100620 |
| H | -2.123787 | -2.864863 | -0.596800 |
| H | -3.998438 | 1.603410 | 0.658367 |
| H | -5.490391 | -0.393622 | 0.521318 |
| C | -1.691723 | -0.775763 | -0.250750 |
| C | -2.224897 | 0.495945 | 0.108412 |
| C | 0.377349 | 0.148242 | -0.443431 |
| C | 1.850488 | -0.008333 | -0.781584 |
| C | 2.696372 | 1.189666 | -0.462607 |
| C | 2.194517 | 2.379308 | -0.116392 |
| C | 0.749346 | 2.617381 | 0.065941 |
| C | -0.149981 | 1.416616 | -0.069568 |
| H | 3.772675 | 1.052631 | -0.583188 |
| H | 2.846535 | 3.238035 | 0.049658 |
| N | -1.427618 | 1.587070 | 0.195360 |
| N | -0.372164 | -0.923519 | -0.532778 |
| O | 0.304198 | 3.715886 | 0.334461 |
| H | 1.934920 | -0.198297 | -1.864004 |
| O | 2.391265 | -2.353444 | -0.868527 |
| N | 2.416652 | -1.195946 | -0.162262 |


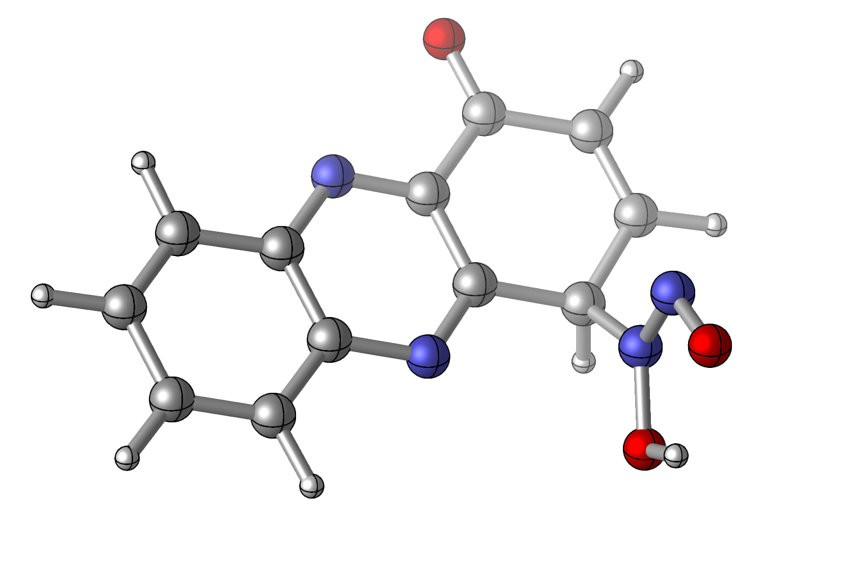


Zero-point correction= 0.194034 (Hartree/Particle)

Thermal correction to Energy= 0.208402

Thermal correction to Enthalpy= 0.209346

Thermal correction to Gibbs Free Energy= 0.150718

Sum of electronic and zero-point Energies= -906.210957

Sum of electronic and thermal Energies= -906.196589

Sum of electronic and thermal Enthalpies= -906.195644

Sum of electronic and thermal Free Energies= -906.254272

**^4^TS_2’-3*_**

E(scf) = -982.787927272 a.u.

ν_min_ = -218.4 cm^-1^

| C | -0.868122 | 0.405606 | -0.064149 |
| --- | --- | --- | --- |
| C | -2.236525 | 0.416613 | -0.150215 |
| C | -0.790328 | 2.820529 | 0.127839 |
| C | -0.135121 | 1.617787 | 0.076877 |
| H | -0.328788 | -0.542404 | -0.103009 |
| H | -2.814093 | -0.502798 | -0.256911 |
| H | -0.255107 | 3.765449 | 0.233656 |
| H | 0.953180 | 1.581774 | 0.143364 |
| C | -2.945799 | 1.650582 | -0.097277 |
| C | -2.210878 | 2.865189 | 0.040740 |
| C | -4.899157 | 2.828782 | -0.118192 |
| C | -6.342464 | 2.900979 | -0.133768 |
| C | -6.975114 | 4.168184 | -0.250821 |
| C | -6.293293 | 5.350211 | -0.138861 |
| C | -4.855666 | 5.395759 | 0.015036 |
| C | -4.157273 | 4.060387 | 0.000882 |
| H | -8.058433 | 4.178369 | -0.396350 |
| H | -6.823960 | 6.302651 | -0.168010 |
| N | -2.841113 | 4.061449 | 0.084937 |
| N | -4.294614 | 1.647698 | -0.170139 |
| O | -4.212517 | 6.440458 | 0.133628 |
| H | -6.569718 | 2.663385 | 1.675360 |
| O | -7.133742 | 1.503939 | -1.920727 |
| N | -7.039684 | 1.745675 | -0.584314 |

Zero-point correction= 0.217651 (Hartree/Particle)

Thermal correction to Energy= 0.233572

Thermal correction to Enthalpy= 0.234516

Thermal correction to Gibbs Free Energy= 0.173648

Sum of electronic and zero-point Energies= -982.570276

Sum of electronic and thermal Energies= -982.554355

Sum of electronic and thermal Enthalpies= -982.553411

Sum of electronic and thermal Free Energies= -982.61427

**^4^3***

E(scf) = -906.362613200 a.u.

| C | -3.798412 | -2.117165 | 0.091887 |
| --- | --- | --- | --- |
| C | -2.428547 | -2.158495 | 0.061642 |
| C | -3.798324 | 0.307559 | 0.059654 |
| C | -4.492363 | -0.873800 | 0.089448 |
| H | -4.369939 | -3.046467 | 0.117514 |
| H | -1.880214 | -3.101554 | 0.061175 |
| H | -4.303651 | 1.274499 | 0.059977 |
| H | -5.583003 | -0.869028 | 0.112980 |
| C | -1.679726 | -0.947324 | 0.024665 |
| C | -2.374964 | 0.297829 | 0.028201 |
| C | 0.313957 | 0.158863 | -0.057357 |
| C | 1.751225 | 0.200542 | -0.100918 |
| C | 2.443841 | 1.433233 | -0.097783 |
| C | 1.794572 | 2.637446 | -0.051327 |
| C | 0.352317 | 2.733126 | -0.013048 |
| C | -0.385316 | 1.420392 | -0.025882 |
| H | 3.534017 | 1.421968 | -0.175789 |
| H | 2.360270 | 3.568918 | -0.081300 |
| N | -1.702286 | 1.470486 | 0.013018 |
| N | -0.331854 | -1.000159 | -0.014106 |
| O | -0.261478 | 3.800455 | 0.016577 |
| O | 2.193660 | -1.979765 | -0.946284 |
| N | 2.476187 | -0.979080 | -0.106902 |
| N | 3.533002 | -1.190313 | 0.611958 |


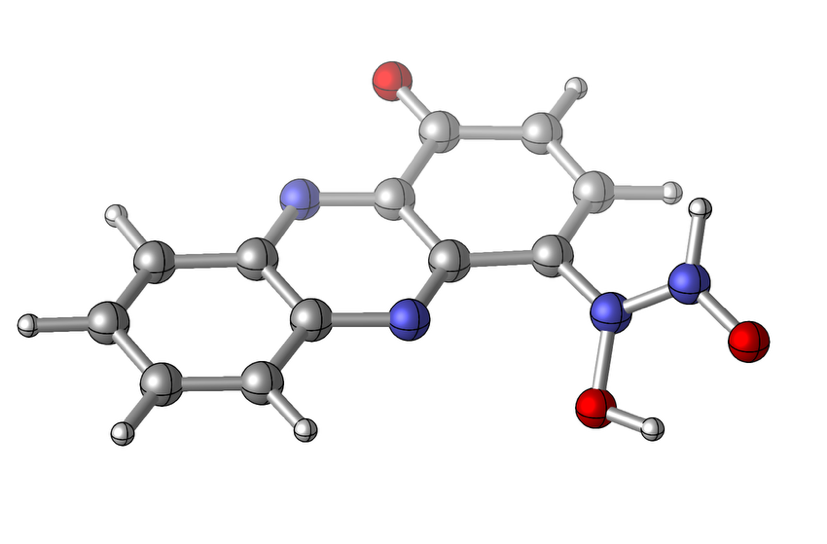


Zero-point correction= 0.194597 (Hartree/Particle)

Thermal correction to Energy= 0.208708

Thermal correction to Enthalpy= 0.209652

Thermal correction to Gibbs Free Energy= 0.152847

Sum of electronic and zero-point Energies= -906.168016

Sum of electronic and thermal Energies= -906.153905

Sum of electronic and thermal Enthalpies= -906.152961

Sum of electronic and thermal Free Energies= -906.209767 9

**^4^TS_3*-4_**

E(scf) = -906.351407104 a.u.

ν_min_ = -580.7 cm^-1^

| C | 0.707285 | -0.548052 | -0.031334 |
| --- | --- | --- | --- |
| C | -0.655741 | -0.577919 | 0.125998 |
| C | 0.710261 | 1.869719 | -0.234639 |
| C | 1.396552 | 0.682197 | -0.212197 |
| H | 1.274591 | -1.479692 | -0.017473 |
| H | -1.198644 | -1.513169 | 0.266599 |
| H | 1.214179 | 2.826885 | -0.372411 |
| H | 2.480297 | 0.674633 | -0.333909 |
| C | -1.386981 | 0.637851 | 0.107316 |
| C | -0.701615 | 1.874325 | -0.075939 |
| C | -3.378361 | 1.783433 | 0.243695 |
| C | -4.820652 | 1.795864 | 0.389687 |
| C | -5.509443 | 3.050610 | 0.344015 |
| C | -4.869757 | 4.235831 | 0.157769 |
| C | -3.422704 | 4.325172 | -0.001592 |
| C | -2.675613 | 3.019796 | 0.049722 |
| H | -6.591215 | 3.039284 | 0.491749 |
| H | -5.424809 | 5.174165 | 0.143515 |
| N | -1.370758 | 3.052481 | -0.103947 |
| N | -2.730440 | 0.625384 | 0.267082 |
| O | -2.831205 | 5.385229 | -0.160649 |
| O | -4.981000 | -0.493069 | 0.731163 |
| N | -5.568630 | 0.684927 | 0.537262 |
| N | -6.800768 | 0.370344 | -0.672114 |

Zero-point correction= 0.190551 (Hartree/Particle)

Thermal correction to Energy= 0.204780

Thermal correction to Enthalpy= 0.205724

Thermal correction to Gibbs Free Energy= 0.148488

Sum of electronic and zero-point Energies= -906.160856

Sum of electronic and thermal Energies= -906.146627

Sum of electronic and thermal Enthalpies= -906.145683

Sum of electronic and thermal Free Energies= -906.20
